# The *sil* Locus in *Streptococcus* Anginosus Group: Interspecies Competition and a Hotspot of Genetic Diversity

**DOI:** 10.3389/fmicb.2016.02156

**Published:** 2017-01-10

**Authors:** Michelle L. Mendonca, Jake C. Szamosi, Anne-Marie Lacroix, Michelle E. Fontes, Dawn M. Bowdish, Michael G. Surette

**Affiliations:** ^1^Department of Biochemistry and Biomedical Sciences, McMaster University, HamiltonON, Canada; ^2^Farncombe Family Digestive Health Research Institute, McMaster University, HamiltonON, Canada; ^3^Department of Pathology and Molecular Medicine, McMaster University, HamiltonON, Canada; ^4^Michael G. DeGroote Institute for Infectious Disease Research, McMaster University, HamiltonON, Canada; ^5^Department of Medicine, McMaster University, HamiltonON, Canada

**Keywords:** *Streptococcus* Milleri Group, *Streptococcus* Anginosus Group, *sil* system, quorum sensing, bacteriocins, cell–cell signaling

## Abstract

The *Streptococcus* Invasion Locus (*Sil*) was first described in *Streptococcus pyogenes* and *Streptococcus pneumoniae*, where it has been implicated in virulence. The two-component peptide signaling system consists of the SilA response regulator and SilB histidine kinase along with the SilCR signaling peptide and SilD/E export/processing proteins. The presence of an associated bacteriocin region suggests this system may play a role in competitive interactions with other microbes. Comparative analysis of 42 *Streptococcus* Anginosus/Milleri Group (SAG) genomes reveals this to be a hot spot for genomic variability. A cluster of bacteriocin/immunity genes is found adjacent to the *sil* system in most SAG isolates (typically 6–10 per strain). In addition, there were two distinct SilCR peptides identified in this group, denoted here as SilCR_SAG-A_ and SilCR_SAG-B_, with corresponding alleles in *silB*. Our analysis of the 42 *sil* loci showed that SilCR_SAG-A_ is only found in *Streptococcus intermedius* while all three species can carry SilCR_SAG-B_. In *S. intermedius* B196, a putative SilA operator is located upstream of bacteriocin gene clusters, implicating the *sil* system in regulation of microbe–microbe interactions at mucosal surfaces where the group resides. We demonstrate that *S. intermedius* B196 responds to its cognate SilCR_SAG-A_, and, less effectively, to SilCR_SAG-B_ released by other Anginosus group members, to produce putative bacteriocins and inhibit the growth of a sensitive strain of *S. constellatus*.

## Introduction

The *Streptococcus* Anginosus/Milleri Group (SAG) is a group of three distinct yet closely related species: *S. anginosus, S. constellatus*, and *S. intermedius*. The species *S. anginosus* is divided into *S. anginosus* subsp. *anginosus* and *S. anginosus* subsp. *whileyi* while the species *S. constellatus* is divided into *S. constellatus* subsp. *constellatus, S. constellatus* subsp. *pharyngis* and *S. constellatus* subsp. *viborgensis* (Jensen et al., 2013). In humans, the SAG lead a dual lifestyle as both commensals and pathogens. These strains can be found asymptomatically colonizing the oral cavity as well as the gastrointestinal and urogenital tracts (Poole and Wilson, 1979; Whiley et al., 1992; Jacobs et al., 1995). However, they are also important pyogenic pathogens involved in empyema and soft tissue abscesses (Ruoff, 1988; Whiley et al., 1992; Coman et al., 1995; Laupland et al., 2006; Ripley et al., 2006; Siegman-Igra et al., 2012; Asam and Spellerberg, 2014) as well as infections of the lower airways (Shinzato and Saito, 1995; Parkins et al., 2008; Sibley et al., 2008). The SAG can be difficult to culture and identify (Ruoff, 1988; Sibley et al., 2010) and are underappreciated pathogens. They have been associated with more invasive pyogenic infections than Group A and Group B *Streptococcus* combined (Laupland et al., 2006; Siegman-Igra et al., 2012).

Host colonization involves competition with resident microorganisms. One mechanism by which streptococci inhibit closely related bacteria is through short peptides called bacteriocins (Dawid et al., 2007). Variable bacteriocin and putative bacteriocin genes are found adjacent to the *sil* locus in GAS, and these are predicted to be under the regulatory control of SilA (Belotserkovsky et al., 2009). The *Streptococcus* Invasion locus (*sil*) system was first identified in Group A Streptococci (GAS), where a transposon insertion in the *sil* system attenuated virulence in a murine model (Hidalgo-Grass et al., 2002). The core signaling system has been characterized and contains the cell–cell signaling peptide SilCR, a peptide processing/export system (SilD/E), and two component sensing system (SilA/B). Upon sensing pheromone peptide SilCR, the histidine kinase SilB phosphorylates the response regulator SilA, upregulating genes involved in SilCR production. These include the SilCR peptide itself and ABC transporters, SilD and SilE. SilC is encoded on the antisense strand of *silCR* and its expression represses SilA-inducible genes in GAS (Hidalgo-Grass et al., 2002; Eran et al., 2007). SilA/B transcriptional regulation is uncharacterized, but induction of the *sil* system is dependent on their presence and expression (Eran et al., 2007). The system also includes a putative CAAX protease, thought to be involved in immunity against or maturation of bacteriocins.

The *sil* system has been implicated in virulence in GAS (Hidalgo-Grass et al., 2002; Salim et al., 2008). Disruption of *silC* leads to attenuation in a murine model (Hidalgo-Grass et al., 2002). As SilC represses SilCR induction, the lack of the pheromone peptide appears to favor pathogenesis. However, interspecies communication occurring between GAS and Group G streptococci (GGS) via SilCR peptide positively regulates SilCR and bacteriocins (Belotserkovsky et al., 2009). Thus, regulation of this system in streptococci is complex and can impact pathogenesis in multiple ways.

The *sil* system has not been characterized in SAG. Here, we describe the *sil* system in 42 SAG genomes, outlining species-specific variation and identification of novel putative bacteriocins. We note this locus as a hotspot for genetic variability with most strains featuring a large cluster of putative bacteriocin/immunity genes. We demonstrate that the inhibitory activity of SilCR-dependent putative bacteriocins can give *S. intermedius* a competitive advantage.

## Materials and Methods

### Strains

Forty-four assembled SAG genomes were used for our analysis (Supplementary Table S1). Seven of these were previously published complete, closed genomes (C1050, C1051, C818, C232, C270, C238, B196; Olson et al., 2013), and 26 were unpublished draft genomes (see below). The strains of these 26 new genomes have been described in Kaiser et al. (2014). The SAG *sil* regions have been deposited in GenBank (accession numbers KY315440-KY315481) (Supplementary Table S1). Eleven draft genomes were also downloaded from NCBI (Benson et al., 2015) (ADME01000005.1, AP013072.1, AJKN01000015.1, AICP01000048.1, AFXO01000004.1, NC 018073.1, AFUP01000001.1, AICQ01000033.1, AFXN01000007.1, AREF01000001.1, AECT01000012.1).

### Assembly and Strain Cluster Assignment

The in-house draft genomes were all sequenced using NexteraXT libraries and Illumina MiSeq using either 150 or 250 bp paired-end reads. The sequences were assembled using our in-house genome assembly pipeline [Surette Lab Assembly Pipeline (SLAP)]. SLAP preprocesses the reads by trimming adapters with Cutadapt (Martin, 2011) and performing quality trimming and error correction with sga preprocess and sga correct (Simpson and Durbin, 2012). It then estimates an optimal kmer size using an in-house adaptation of the program Kmergenie (Chikhi and Medvedev, 2014), and calls four separate assemblers [MaSuRCA (Zimin et al., 2013), IDBA (Peng et al., 2011), SPAdes (Bankevich et al., 2012), and Velvet (Zerbino and Birney, 2008)] to produce four candidate assemblies. The assemblies are then scaffolded using SSPACE (Boetzer et al., 2011) and quality metrics are provided to the user via FastQC (Andrews, 2010) for read quality and QUAST (Gurevich et al., 2013) for assembly quality. For our purposes, the best assembly was chosen for each strain based on N50 value and total assembly length. All in-house code is available from the authors upon request.

We assigned our samples to strain clusters following Jensen et al. (2013). We found each of the seven housekeeping genes used in the paper in all of our strains. The SAG accession numbers for *map* (KY274883 – KY274924), *pfl* (KY274925 – KY274966), *ppaC* (KY274967 – KY275008), *pyk* (KY275009 – KY275050) *rpoB* (KY275051 – KY275092), *sodA* (KY275093 – KY275133), and *tuf* (KY275134 – KY275175) are as indicated. All genes were present in all samples with the exception of *sodA*, which was absent from C1051. These genes were aligned with the trimmed sequences used by Jensen et al. (2013), trimmed to the same length, and a phylogenetic tree was constructed using MEGA 7.0.20 using default parameters with the following exceptions: we used a minimum-evolution model with 500 bootstrap replicates and pairwise deletion for missing data.

### Identifying the Putative *sil* Region in SAG

The putative *sil* system was identified in the published genome of *S. intermedius* B196 (Accession number: NC_022246.1) based on its structural similarity to the GAS and GGS *sil* locus. We used megablast (Altschul et al., 1997) to find each gene from this putative *sil* locus in the 44 additional SAG genomes. We found the genes together on a single contig in 42 genomes (aside from *S. intermedius* B196) and split across multiple contigs in two genomes (M1 and C424, which we excluded from our analysis). A list of the 42 strains used for our analysis is provided in Supplementary Table S1. The 42 putative *sil* regions were annotated from the BLAST hits using in-house Python and Biopython scripts (van Rossum and Drake, 2001; Cock et al., 2009) with manual adjustments, and the annotations were visualized and compared using Geneious (Kearse et al., 2012).

### Phylogenetic Trees

A phylogenetic tree of the *sil* locus was generated using MrBayes (Huelsenbeck and Ronquist, 2001; Altekar et al., 2004). Each gene (*silA* through *silD*) was aligned individually using MUSCLE (Edgar, 2004) and the alignments were concatenated (with gaps in place of the nucleotides for missing genes), providing a single alignment of all genes. The genes were each assigned their own partition in the MrBayes model, with a 4-by-4 General Time Reversible evolutionary model (Tavare, 1986) and invariant/gamma rates distribution (Yang, 1994) for each partition. Since many sequences were missing individual genes, the presence or absence of each gene was encoded as binary “standard” data in a separate partition (Lewis, 2001). The trees were run until the average standard deviation of the split was less than 0.01 and the consensus tree was visualized using the Interactive Tree of Life (iTOL) (Letunic and Bork, 2016).

### Annotating the Bacteriocin Region

The putative bacteriocin region adjacent to the *sil* region was annotated using the online version of antiSMASH (Blin et al., 2013, 2014) accessed November of 2013. Only the results of the antiSMASH-internal GLIMMER (Delcher et al., 1999) annotation were used. In order to determine how variable the accessory regions are, the sequences from the GLIMMER hits were translated and preliminary clustering was undertaken using OrthoMCL (Fischer et al., 2011). This produced 16 putative orthologous groups, here referred to as ORF1 through ORF16. To identify any pseudogenes or related ORFs not discovered by GLIMMER, we manually divided each cluster into smaller groups based on the MUSCLE alignment of the cluster (if multiple groups were apparent) and generated a consensus sequence for each group. We searched naively for each of these consensus sequences in all of the bacteriocin regions (tolerant to 20% mismatch at the amino acid level).

We generated a tree for each of the 16 ORF clusters produced by OrthoMCL using FastTree (Price et al., 2009). The location of each putative accessory gene in each strain was marked and patterns of synteny were manually examined.

We searched for each ORF in the non-redundant protein sequences (nr) database with Blastx (Altschul et al., 1997) to identify putative functions. In addition, each ORF was searched against the BACTIBASE database (Hammami et al., 2007, 2010) of bacteriocins to identify hits with existing bacteriocins.

### Bacterial Culturing Conditions

We cultured *S. intermedius* B196 and *S. constellatus* M505 on Todd Hewitt agar with 0.5% yeast extract (THY) and incubated at 37°C in a 5% CO_2_ incubator for 3 days. We inoculated THY broth with colonies and incubated either at 5% CO_2_ or anaerobically (5% CO_2_, 5% H_2_, 90% N_2_) at 37°C overnight. We used broth cultures to conduct further experiments. THY supplemented with 75 μg/ml spectinomycin (THY-spec) was used to grow our knockout strain (see below). We used *Escherichia coli* Top10 chemically competent cells (Life Technologies) during cloning. *E. coli* carrying desired knockout constructs grew on Luria-Bertani agar with 100 μg/mL spectinomycin (LB-spec).

### Identification of SilA Binding Sites in *S. intermedius* B196

Binding of response regulator SilA to direct repeats in GAS and GGS affects expression of *sil* locus components as well as putative bacteriocins (Hidalgo-Grass et al., 2002). The SilA binding site in GAS (ACCATTCATG-11bp-ACCTTTTAAG) (Belotserkovsky et al., 2009) was used as a query to identify putative sites in *S. intermedius* B196 using the motif search in Geneious (Kearse et al., 2012). The ClustalW 2.1 alignment of these sites (Goujon et al., 2010) was used to generate an *S. intermedius* B196 consensus sequence, visualized using WebLogo (Crooks et al., 2004).

### SilCR Knockout Construction

We constructed a deletion mutant of *silCR* in *S. intermedius* B196. First, we cloned the spectinomycin resistance marker from pDL278 (Dunny et al., 1991) along with its promoter into pUC19 with primers specF and specR as shown in **Table 1**. We then cloned the upstream and downstream regions of *silCR* in *S. intermedius* on either side of a spectinomycin resistance marker in pUC19 using primers SilCRupF, SilCRupR, SilCRdownF, and SilCRdownR (**Table 1**). We amplified the cassette (*silCR* upstream: *specR*: *silCR* downstream) using PCR with primers SilCRupF and SilCRdownR and purified it. Our laboratory has found that *S. intermedius* B196 is naturally competent and can be transformed using the competence stimulating peptide, ComC (DSRIRMGFDFSKLFGK) (data not shown). An overnight THY broth culture was diluted 1000 fold in 500 μL THY and incubated for 2 h at 37°C in 5% CO_2_ before adding competence peptide (10 ng) and purified cassette DNA (500 ng). The reaction was incubated in normal growth conditions for an hour before plating the transformation reaction on THY- spec. We incubated the plates for 2 days anaerobically at 37°C and screened colonies using PCR and sequencing to verify the deletion.

**Table 1:** Primers used for *silCR* knockout construction and Real time PCR.

Knockout construction		
Primer	Sequence (5′–3′) with restriction sites underlined	Restriction enzyme
specF	AAAAAGGATCCGACGAAGAGGATGAAGAGG	*Bam*HI
specR	AAAAAGTCGACCCCAAATATTAAATAATAAAAC	*Sal*I
SilCRupF	AAAAAGAGCTCCAAAAAGCAATACTGAAGT	*Sac*I
SilCRupR	AAAAGGATCCCATGTATTATTATAACCCGAC	*Bam*HI
SilCRdownF	AAAAGTCGACCTAAAAATACAAATAACCTCATTG	*Sal*I
SilCRdownR	AAAAGCATGCCTTCTCTCTATTTTTACATCAG	*Sph*I

**Real time PCR**		

**Primer**	**Sequence (5′–3′)**	

SilCRF	ACAAACATTGGATCATTTTCGTACACTCAC	
SilCRR	AGCCTAATGTATTATTTGAATCTCCCTTTGTTAAAC	
SilEF	GAATTACCAATGTCTTTTTTTGCTACGAGACG	
SilER	GTCAAGAAAGAGTGAAAGTATTGTAGAAGCG	
ORF1F	CTCACTCGAAAAATTTGAAGTACTGAACTCC	
ORF1R	CCATGTTGCGGCTAAACAAGTATAATGTG	
ORF16F	TGAATACAAAAACATTGGAAAAATTTGAAGCACTG	
ORF16R	GGTCTTGATTCCTAATCTCAATCCATTACCTG	
SodAF	CGGCAAGGCTTTAGAACAACTTTTAGC	
SodAR	CTTCTGGTGTCATCAATTCCCAAAAAAGAC	
DnaKF	AAGTTATCTTGGTTGGTGGG	
DnaKR	AGGGTTTACTGATTTGTTTGG	
RecAF	CTGCATCTATCAATAAAACGAAGAC	
RecAR	AGCGAACTGAAGCATAAAAC	

### Bacteriocin Activity Assays

To assess bacteriocin activity, we adapted top-agar overlay experiments (Kormin et al., 2001; Maricic and Dawid, 2014). *S. intermedius* B196 (the bacteriocin producer) and mutant strain were grown in an overnight anaerobic broth culture as previously described. A volume of 4 μL of the overnight culture was spotted on THY agar and incubated in the anaerobe chamber at 37°C for 2 days. In some cases, synthetic SilCR peptide was spotted along with culture. SilCR peptide was synthesized by RS Synthesis, LLC (Louisville). The amount of peptide added was dependent on the experiment. *S. constellatus* M505, which lacks the bacteriocin cluster and *caax* gene, was used as a bacteriocin-sensitive strain. This was grown anaerobically in THY for 24 h. Top agar (THY) was prepared using 1.5% agar as this higher percentage improved the visualization of the zone of clearing. A 100 μL of overnight broth culture of *S. constellatus* M505 was added to 5 mL molten Top agar and inverted three times before pouring onto the agar plates with *S. intermedius* spots. Plates were incubated for 1–2 days at 5% CO_2_ at 37°C.

### Relative Real Time PCR

To assess SilCR dependent expression of putative bacteriocins, we designed primers for specific genes in the *sil* locus and reference genes (molecular chaperone *dnaK* and recombinase *recA*) (**Table 1**). Biological replicates of *S. intermedius* B196 and mutant were grown and spotted on THY agar as described, with and without synthetic SilCR peptide. After 16 h of growth in the anaerobic chamber at 37°C, bacterial spots were resuspended in RNAlater Solution (Ambion). Bacterial RNA was purified using enzymatic lysis, TRIzol (Invitrogen) and the RNeasy mini kit (Qiagen) as described in Fei et al., 2016. To eliminate DNA contamination, samples were treated with the RNase free DNase set (Qiagen) and subsequently purified using the RNeasy mini columns (Qiagen). RNA was normalized using readings from the Nanodrop prior to cDNA preparation using the SuperScript^®^ III first-strand cDNA synthesis system for RT-PCR (Invitrogen) as per manufacturer’s instructions. SsoFast^TM^ Evagreen^®^ Supermix (Bio-Rad) was used for real time PCR as per manufacturer’s instructions. Real-time PCR was conducted on the CFX96 Touch^TM^ Real-time PCR detection system (Bio-Rad). Thermal cycling conditions included an initial 95°C for 30 s and 39 cycles of 95°C for 5 s and 55°C for 5 s. The gene expression level of sample groups was normalized to wild type *S. intermedius* B196 without SilCR peptide and calculated using the ΔΔCT method in CFX manager (Bio-Rad).

## Results

### Identification of the *sil* System in *S. intermedius* B196

**FIGURE 1:**
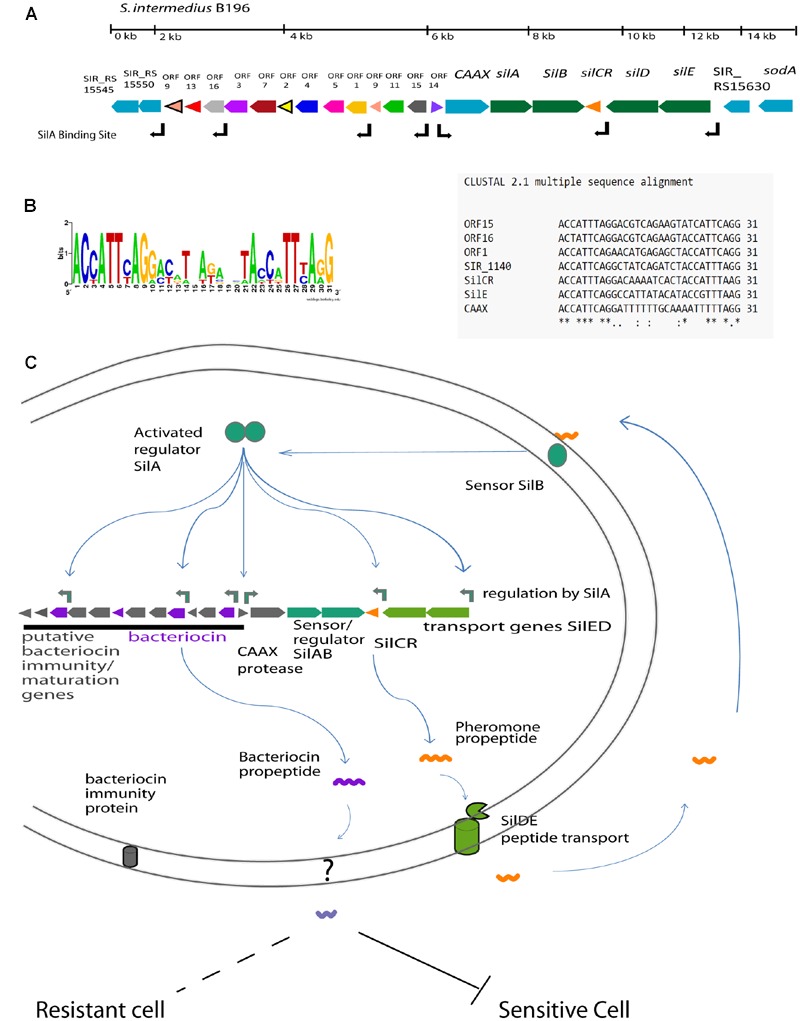
**The predicted Sil system in *Streptococcus intermedius* B196. (A)** The organization of the *sil* locus showing the signal peptide gene (*SilCR*) and signaling transduction genes (*silAB* and *silED*) in green. The associated locus genes including putative CAAX protease is shown in blue and bacteriocin associated gene clusters are indicated in color. The positions of predicted SilA binding sites are as indicated. **(B)** The predicted SilA binding site and consensus sequence. **(C)** Schematic illustration of the *sil* regulatory network in the *Streptococcus* Anginosus Group based on the *sil* system in Group A *Streptococcus* (Eran et al., 2007; Belotserkovsky et al., 2009).

We have previously sequenced and annotated the genome for the clinically isolated strain *S. intermedius* B196 (Olson et al., 2013). We identified a region with structural similarity to the *sil* region previously identified in GAS and GGS. This region contains five of the six genes included in the GAS/GGS *sil* locus (*silA, silB, silCR, silD*, and *silE*), with the same relative positioning and orientation (**Figure 1A**). The locus is bounded by a putative D-Ala-D-Ala carboxypeptidase (SIR_RS15545) at one end and *sodA* at the other. Adjacent to the carboxipeptidase is a conserved hypothetical protein (SIR_RS15550) that has a thioredoxin-like domain. This is followed by 13 small ORFs containing putative bacteriocins, followed by a gene with homology to CAAX proteases. Downstream of the CAAX protease are the *sil* genes including *silA, silB, silCR, silD*, and *silE*. We did not find evidence for a counterpart to *silC* in *S. intermedius* B196 based on nucleotide similarity to GAS *silC*. While there is an open reading frame (ORF) on the antisense strand of SilCR, there is no evidence that it is transcribed or plays any role in *sil* regulation. More research is required to conclude if this is *silC*.

Comparison between the *S. intermedius* B196 and GAS *sil* systems revealed sequence divergence despite the overall similar organization of the *sil* loci. Although, the putative *sil* region in *S. intermedius* B196 shares its structure with that found in *Streptococcus pyogenes* AF493605.1 (Hidalgo-Grass et al., 2002), these genes have low rates of sequence identity. *S. intermedius* B196 SilA is only 49.4% identical (85.7% similar) to GAS SilA in amino acid sequence (60.0% nucleotide identity). B196 SilB is 34.6% identical and 73.9% similar to GAS SilB (53.7% nucleotide identity). B196 SilCR is 28.0% identical and 68.0% similar to GAS SilCR (47.8% nucleotide identity). B196 SilD is 56.2% identical and 90.3% similar to GAS SilD (63.6% nucleotide identity). Finally, B196 SilE is 74.5% identical and 88.6% similar to GAS SilE (68.5% nucleotide identity).

In other streptococci, the *sil* system and putative bacteriocins are transcriptionally regulated by response regulator SilA and the SilCR pheromone peptide (Hidalgo-Grass et al., 2002). The SilA binding site in GAS (ACCATTCATG-11bp-ACCTTTTAAG) was used to find putative SilA binding sites in the *S. intermedius* B196 *sil* locus, as highlighted in **Figure 1A**. Direct repeats were found upstream of *silCR, silE, caax, SIR_RS15550* and in the accessory region upstream of *ORF1, ORF15*, and *ORF16*. The predicted operator was conserved in all of these genes except CAAX protease, which had inconsistencies in the second repeat (**Figure 1B**).

A schematic for the hypothetical regulation of the *sil* system in SAG is shown in **Figure 1C**. The predicted regulatory network functions as follows. SilCR is produced by SAG species and exported. Sensing of extracellular SilCR peptide induces SilB to autophosphorylate and phosphorylate response regulator SilA. SilA, in turn, induces expression of the seven genes indicated in **Figure 1B**. Induction of these genes is predicted to amplify the response via SilCR dependent autoregulation and induce production of bacteriocins which can inhibit closely related bacteria.

### Bacteriocin Activity in *S. intermedius* B196 is Regulated by SilCR

**FIGURE 2:**
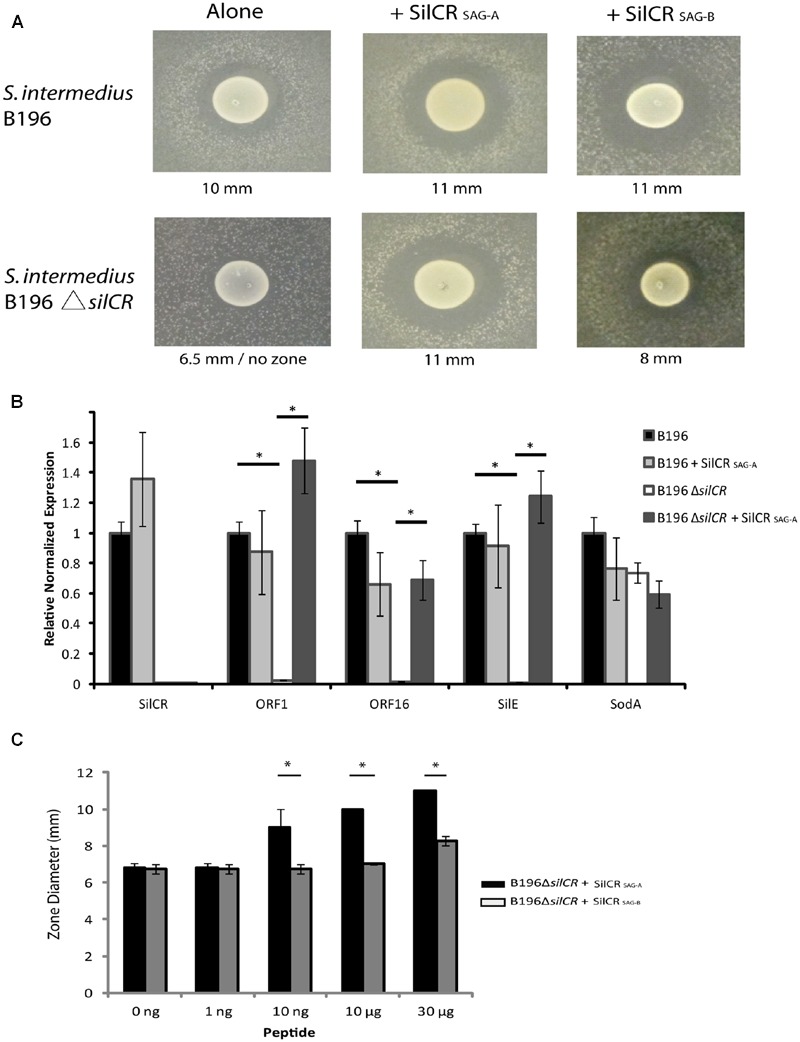
**Bacteriocin-mediated competition in *S. intermedius* is controlled by the SilCR peptide.** The gene for the pheromone peptide SilCR was deleted in *S. intermedius* B196 (*S. intermedius* B196 Δ*silCR*). **(A)** Competition in the wild type and mutant was ascertained using an overlay of sensitive strain *S. constellatus* M505. The two identified SAG SilCR peptides were exogenously added to wild type and mutant and the diameter of the inhibition zone measured as shown. **(B)** Relative reverse transcription PCR on B196 and B196Δ*silCR* for experiment shown in **(A)** without the M505 overlay (^∗^*p* < 0.005). **(C)** A concentration dependent induction of bacteriocin activity by SilCR peptides A and B in *S. intermedius* B196. Note the B196 colony diameter is ∼6.5 mm. SilCR_SAG-A_ induced zones are significantly larger than those with SilCR_SAG-B_ (^∗^*p* < 0.005).

We investigated the role of the *sil* system in inter-species competition using *S. intermedius* B196 as a model. A putative SilA binding site was found upstream of putative bacteriocins ORF1, ORF15, and ORF16 (**Figure 1**). To investigate whether SilCR regulates bacteriocin expression in *S. intermedius* B196, we constructed a *silCR* deletion mutant. We assayed bacteriocin activity by spotting *S. intermedius* B196 and its mutant on THY agar and after growth, applying a top agar overlay of a sensitive strain, *S. constellatus* M505. Strain M505 was chosen because it lacks the *sil* accessory region (see below) and we therefore predicted it would lack immunity genes and be sensitive to SilCR-dependent bacteriocins produced by B196. When M505 was overlaid in top agar over B196, a clear zone of growth inhibition was observed (**Figure 2A**). This activity is lost in the B196 Δ*silCR* mutant (*S. intermedius* B196 Δ*silCR*; **Figure 2A**). Exogenous addition of the synthetic SilCR peptide from B196 (SilCR_SAG-A_) restored the wild type phenotype, demonstrating that SilCR induces production of an inhibitor of strain M505.

In order to identify whether deletion of *silCR* affected gene expression of the putative bacteriocins ORF1 and ORF16, we conducted relative real-time PCR on the wild type and mutant strains (**Figure 2B**). We used spotted B196 and B196 Δ*silCR* with and without exogenously added SilCR_SAG-A_ for our analysis. Deletion of *silCR* downregulated expression of gene *silE* and putative bacteriocins *ORF1* and *ORF16* in the samples tested. Addition of SilCR _SAG-A_ to the knockout induced expression of *ORF1, ORF16* and *silE* by 61-, 43-, and 138-fold respectively; demonstrating that SilCR regulates expression of putative bacteriocins *ORF1* and *ORF16* and these are putatively involved in inhibition of *S. constellatus* M505.

### Comparative Genomic Analysis of the *sil* Locus Separates SAG Strains into Two *silCR* Peptide Groups

**FIGURE 3:**
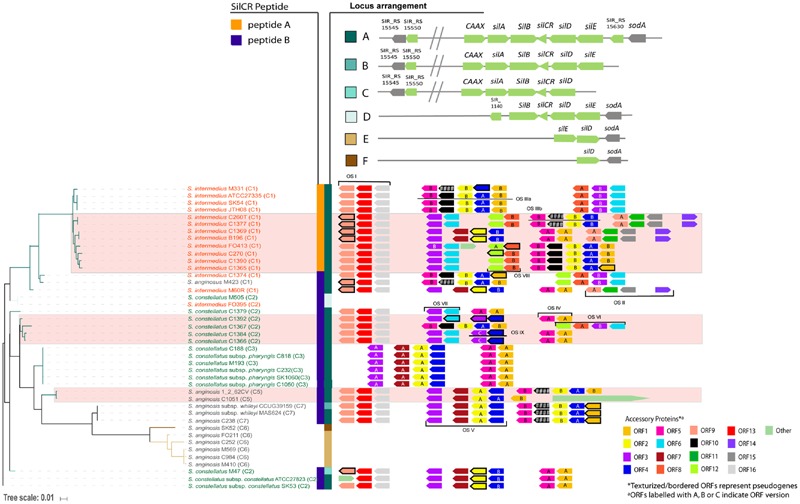
***sil* locus heterogeneity in the *Streptococcus* Anginosus Group (SAG).** A tree was constructed based on *sil* genes in 42 strains. Six locus arrangements were found (shown in locus arrangement legend with the locus annotation). Hashes (//) in the annotation indicate a putative bacteriocin accessory region. Two SilCR peptides were identified, shown in the peptide legend. SAG species text is color coded (*S. intermedius* in orange, *S. anginosus* in gray, and *S. constellatus* in green). The accessory region ORFs are shown with the tree. Some ORFs have several versions, indicated by the letter inside the ORF. Genes that have acquired mutations leading to pseudogenes in some strains and are indicated in the figure.

We investigated the distribution of the *sil* system in SAG. Forty four SAG strains were used in our analysis: a combination of in-house sequenced genomes and those available online. Only genomes containing the *sil* genes on a single contig were analyzed (42 of the 44 genomes; as listed in Supplementary Table S1). We used the multilocus sequence analysis (MLSA) described by Jensen et al. (2013) to assign our strains into seven designated clusters for SAG. Our MLSA tree reproduced the clusters found by Jensen et al. (2013) with our new strains neatly nested within six of the seven clusters. Strain cluster assignments are included in **Figure 3** and the MLSA phylogeny is shown in Supplementary Figure S3.

Initial investigation of the *sil* sequences divided SAG strains into two groups. In 24 of the 42 genomes, both *silB* and *silCR* appeared truncated; *silB* at the 5′ end and *silCR* at the 3′ end. Further examination, however, found that this 5′ end of *silB* was well-conserved in 22 of the 24 strains. In addition, results from a BLAST search of this region showed high identity with histidine kinases, which is consistent with the putative function of SilB. Indeed, the gene had been annotated as such in at least one of the published genomes in our data set (protein accession YP 008494978 from genome C232), implying that we had found a variation of *silB*. These 22 strains also had a well-conserved region following their truncated *silCR*. A stop codon located 150 bp from the start of *silCR* was used to define the variation of putative *silCR*. Thus, we identified two distinct SilCR peptides in SAG, SilCR_SAG-A_ and SilCR_SAG-B_ (previously truncated version), with predicted mature amino acid sequences GWLEDLLKHFSGYNSLTKGDSNNTLG and GWLEDLFSPYLKKYKLGKLGQPDLG, respectively. Notably, each SilCR peptide allele associated exclusively with a corresponding variant of histidine kinase SilB. This suggests that the response by a strain could be dependent on the kinase variant. The conserved 5′ end of SilCR propeptide is hypothetically cleaved at the double glycine residues to produce the mature SilCR peptide.

To investigate whether *S. intermedius* B196 could respond to the alternative peptide, SilCR_SAG-B_ was added to the knockout (**Figure 2A**). Despite high peptide concentrations, SilCR_SAG-B_ induced a smaller inhibitory zone in M505 than did SilCR_SAG-A_ (8 mm vs. 11 mm). To further test the peptide dependent bacteriocin induction of strain B196, several concentrations of each peptide were used. We found that higher concentrations of SilCR_SAG-B_ were required to produce inhibitory zones comparable to those produced by SilCR_SAG-A_ (**Figure 2C**). Thus, while strain B196 can sense and respond to both pheromone SilCR peptides produced by SAG strains, its inhibitor production is peptide- and concentration- dependent requiring high concentration of the non-cognate signaling peptide to elicit a response.

To investigate the phylogeny of the *sil* locus in the 42 SAG genomes, the gene presence, sequence, and orientation were used to construct a tree (**Figure 3**). The tree of *sil* genes corresponded well with SAG species. Six different organizations of the *sil* locus were noted in SAG, as depicted in the locus arrangement legend in **Figure 3**. The type of SilCR peptide the strain produced (SilCR_SAG-A_ vs. SilCR_SAG-B_) is also indicated in the figure. Strains encoding SilCR_SAG-A_ have low diversity overall and are monophyletically nested within *S. intermedius*, while all three species can encode SilCR_SAG-B_. In 32 of the 42 *sil* loci, we identified the canonical arrangement of the *sil* locus (locus arrangement A in **Figure 3**). This arrangement is found in strains carrying SilCR_SAG-A_ and SilCR_SAG-B_ and is the arrangement seen in *S. intermedius* B196 (**Figure 1A**). The additional *sil* locus arrangement groups are characterized by the loss of genes and when SilCR is present, only included SilCR_SAG-B_. Locus group B is defined by an intact *sil* region but is altered next to *silE* with *sodA* absent and includes a single *S. anginosus* strain. The other arrangements of the *sil* region (C-D) are represented by deletions leading to loss of core *sil* genes. Group C consists of a single *S. constellatus* strain and is defined by missing *silE* and *sodA*. Group D is represented by one *S. constellatus* strain and one *S. intermedius* strain and is characterized by missing a number of *sil* locus components including the putative bacteriocin region, CAAX protease and *silA* response regulator. Group E is composed of only *S. anginosus* strains, and is characterized by the lack of all *sil* genes and accessory proteins except *silD, silE*, and *sodA*, with an inversion in *silD* and *silE* relative to *sodA*. Group F includes a single *S. anginosus* strain and is identical to Group E but missing *silE*. Overall, the *S. anginosus* strains had the most variation in composition of the *sil* locus with all locus groups represented except Groups C and D.

We identified a few strains where one or more *sil* genes had undergone inactivating mutations. A number of these pseudogenes appear to be the result of a few single mutation events and are consistent with the *sil* tree (**Figure 3**). In strains SK54, ATCC27335, and JTH08 (sister taxa in our *sil* gene phylogeny), *silCR* has a single-nucleotide deletion at base 12. In strains C1366 and C1384 (also sister taxa), *silCR* has a different single-nucleotide deletion at base 14. The *sil* system is otherwise intact in these strains. In strains C188, C232, and C818 (three of six sisters in an unresolved node in our phylogeny), the secretion protein *silD* has a single-nucleotide deletion at base 1314. These strains also have otherwise-intact *sil* regions. In addition to these, a small number of individual strains do not share *sil* gene inactivation events with other strains in our data set. Strain M410 has a partial *sil* region (containing *silE, silD*, and *sodA* only) and a single nucleotide deletion at base 972 in *silE*. Strain CCUG39159 has an intact *sil* region and an SNP that introduces a stop codon at base 1342. Strain M47 appears to have undergone a larger genome rearrangement event, with a 70 Kb region intervening between partial *silD* and *silE* genes. Strains M423, 1_2_62CV, and C270 have different frameshift mutation events in non-*sil* genes (SIR_RS15550 for the first, CAAX for the latter two). Finally, strain SK1060 appears to be degenerate in several places; the SIR_RS15550 homolog contains a single nucleotide insertion at base 95, while CAAX, *silA, silB*, and *silD* contain deletions at bases 558, 18, 136, and 1107 respectively.

### Heterogeneity in the *sil* Accessory Bacteriocin Region Implies Strain Specific Competition

The *Streptococcus sil* system controls expression of competitive bacteriocins in GAS (Eran et al., 2007; Belotserkovsky et al., 2009; Armstrong et al., 2016). In *S. intermedius* B196, we observe that bacteriocin activity is dependent on this signaling system. We discovered a highly variable accessory region in all strains carrying *sil* locus groups A-C (34 of the 42 genomes). We investigated the hypothetical ORFs in this region for putative bacteriocin and immunity genes. Sixteen distinct orthologous groups were identified using OrthoMCL. A detailed similarity comparison within each orthologous group is included in the Supplementary Similarity Files. The amino acid identity heatmap for each ORF is shown in the Supplementary Figures. In general, while some ORFs are highly conserved within SAG, others are not. For example, ORF13 is conserved with 97% or higher amino acid identity across 28 strains while ORF2 is clearly divided into two groups with one group having 44% amino acid identity to the other. Using this classification scheme, it was apparent that some strains had two copies of predicted ORFs (e.g., ORF4 in *S. anginosus* 1_2_62CV; **Figure 3**). It is important to note that the two copies are not identical and, given their patterns of synteny, it is not likely that they are functionally equivalent.

We assigned putative functions to the 16 ORFs identified based on similarity to hits in the non-redundant protein sequence database using BlastX (NCBI). These are summarized in **Table 2**. In total, six putative bacteriocins were identified (ORF1, ORF2, ORF6, ORF8, ORF15, and ORF16). Of the six bacteriocins, two were highly similar to existing bacteriocins (ORF2 is similar to bovicin 255 and ORF15 is similar to ThmA bacteriocin). The non-bacteriocin ORFs may have chaperone or immunity functions. ORFs 4 and 7 had high similarity with a bacteriocin secretion protein and a bacteriocin immunity gene, respectively. ORF3 has a putative role in replication. ORF14 shares some nucleotide identity with a tRNA (cytidine/uridine-2′-*O*-)- methyltransferase. It is unknown whether ORF14 serves a similar function in SAG. Six of the putative ORFs did not have predicted functions (ORFs 5, 9, 10, 11, 12, and 13). Characterization of these genes is required to gain insight into their role. However, some speculation based on the patterns of co-occurrence with other genes is possible. Several of the strains have acquired mutations in some of these accessory genes that has led to pseudogenes. These include two predicted bacteriocin genes (ORF1 and ORF2), several genes with no assigned function (ORF3, ORF9, ORF10, and ORF12) and one gene assigned to bacteriocin secretion function (ORF4). No pseudogenes were detected in any of the predicted immunity genes.

**Table 2:** Bioinformatic analysis of identified putative bacteriocin accessory region ORFs using a sequence search against the non-redundant protein sequence database using BlastX (NCBI).

ORF	BlastX best hit	Cover	Identity	Score	E-value	Accession
1	Bacteriocin; Class IIc	99%	99%	145	3e-43	WP_006267535.1
2	Bovicin 255 variant; Class II	71%	62%	85.9	2e-20	AAR02622.1
3	Role in replication	97%	67%	119	4e-32	WP_030127173.1
4	Bacteriocin secretion protein	57%	37%	46.2	7e-04	EWM55695.1
5	Hypothetical protein	98%	88%	145	5e-43	WP_020998893.1
6	Bacteriocins class II with double glycine leader peptide	88%	54%	67.8	1e-13	KEQ38561.1
7	Bacteriocin immunity protein	98%	99%	194	2e-61	WP_006267076.1
8	Bacteriocin class II with double glycine leader peptide family protein	88%	94%	137	4e-40	WP_009568291.1
9	No hits					
10	No hits					
11	Hypothetical protein	98%	100%	62.4	5e-11	WP_021002964.1
12	No hits					
13	Hypothetical protein	84%	53%	42.7	3e-4	EWM55551.1
14	tRNA (cytidine/uridine-2′-*O*-)- methyltransferase TrmJ	50%	46%	37.4	0.39	WP_059746670.1
15	ThmA bacteriocin; Class IIc	98%	63%	82	7e-19	KIQ75182.1
16	Bacteriocin BlpU; Class IIc	72%	70%	76.6	5e-17	WP_021143664.1

There were some clear patterns of synteny in the accessory regions of the *sil* loci (**Figure 3**). These became more apparent after taking the subclusters of each ORF into consideration. We describe the syntenic groupings of ORFs as “ORF sets” (OS) because the operon structures have not been established for these strains. In combination with our putative functional assignments, we attempted to characterize these ORF sets (Supplementary Figure S1) in order to group bacteriocins with co-occurring genes that may be functionally associated with them. All ORF sets except OS IX contain at least one putative bacteriocin (Supplementary Figure S1). Three ORFs occurred only once each across all strains and are labeled “other” (**Figure 3**). Putative bacteriocin ORF1A is exclusively found adjacent to ORF5A, while ORF1B is separated from ORF5B by bacteriocin ORF2 and its associated genes. The two versions of putative bacteriocin ORF2 were found in two distinct clusters, OS III and OS V. ORF2 is always associated with a version of the putative secretion protein, ORF4. It is also always adjacent to either the putative immunity gene ORF7 or the unknown ORF10, suggesting an immunity function for the latter. Putative bacteriocin ORF6 is always found adjacent to ORF3, implying ORF3 may confer immunity against ORF6. A version of ORF3 is also found associated with ORF2 in OS V. It may be that ORF3, rather than ORF7, confers immunity to ORF2A. Putative bacteriocin ORF8 was found adjacent to ORF12 in most strains (although ORF12 has been mutated to a pseudogene in some of these strains). ORF12 was not present in strains lacking ORF8 and may therefore be only required in the presence of ORF8. Uncharacterized ORF9 is associated with two putative bacteriocins (ORF15 and 16) in two separate ORF sets (OS I and OS II). In each case, the genes are also associated with a hypothetical protein of unknown function (ORFs 11 and 13, respectively). None of the strains carried all six putative bacteriocins, implying that each strain may be susceptible to at least one bacteriocin. A number of *S. intermedius* strains had five predicted bacteriocins (**Figure 3**) implying that this species has high competitive potential. *S. constellatus* C1367 and *S. anginosus* C423 also had five predicted bacteriocins.

The arrangements of ORF sets within the accessory regions correspond imperfectly with the *sil* tree, indicating a large amount of recombination or horizontal gene transfer is occurring within this region. OS I is found in a majority of strains. The six strains lacking OS I form a monophyletic group with low genetic distance in the *sil* region, indicating a recent loss event. Areas of apparent recombination are highlighted with pink boxes in **Figure 3**. OS I occurs at one end of the bacteriocin accessory region in the strains where it is present. OS II is found on five genomes, in two positions relative to other ORF sets. OS IV typically appears at the downstream end of accessory regions where it is found (except in strains C1369 and B196), while OS III may be at the downstream end or may be upstream of OS VI. OS V occurs either upstream of OS II or OS III. In either case it is directly downstream of OS I.

## Discussion

In GAS and GGS, the *Streptococcus sil* system senses and responds to pheromone peptide SilCR with induction of endogenous SilCR production and expression of bacteriocins. In some streptococci, expression of *silCR* is inhibited by expression of *silC*, which is encoded on the antisense strand at the 3′ end of *silCR*. In those organisms, it has been shown that *silC* expression can repress SilCR-activated promoters (Hidalgo-Grass et al., 2002; Eran et al., 2007). No conserved *silC* ORF across all *silCR*+ strains of SAG was found and more research is required to identify if there is a *silC* variant in SAG.

The prevalence of the *sil* locus within GAS is low, with only 4 out of the 19 fully sequenced genomes of GAS carrying it. Remnants of the locus have been left behind in strains that do not (Kizy and Neely, 2009; Michael-Gayego et al., 2013; Jimenez and Federle, 2014). The prevalence is higher in GGS, with all sequenced strains carrying a functional SilCR gene (Belotserkovsky et al., 2009; Michael-Gayego et al., 2013). Our analysis of SAG showed that the majority of the strains sequenced carry the locus. Locus arrangement Groups A and B in **Figure 3** shows that all components of the locus are present in most SAG strains, although individual genes do appear to be degenerate in some strains. The other groups included few strains and, like GAS, showed loss of some *sil* genes. Further study is needed to determine whether the lack of a fully functional *sil* system affects a strain’s competitive fitness or ability to colonize or invade.

In SAG, considerably higher genetic heterogeneity was seen in the bacteriocin accessory region than in the *sil* gene region. *S. anginosus* displayed the most variation in the presence of *sil* genes, implying that the locus may not be under stabilizing selection and may not be required for competition in this species. Conversely, most *S. intermedius* and *S. constellatus* strains were included in locus group A, suggesting that the *sil* locus may be more competitively necessary in these species, at least in the clinical context from which these strains were isolated.

The bacteriocins identified in the SAG *sil* locus have not been characterized or directly associated with intra- or interspecies competition; however, the identification of six putative bacteriocins within the *sil* locus in SAG is a new finding. Bacteriocins can mediate competition with closely related bacteria and can provide a competitive advantage during *in vivo* colonization experiments (Dawid et al., 2007). ORF2 is a homolog of bovicin 255, which has been shown to inhibit growth of select streptococci (Whitford et al., 2001). ORF15 is homologous to thermophilin 13 bacteriocin (ThmA), which is produced by *S. thermophilus* and can inhibit a broad range of Gram-positive bacteria including spore formers (Marciset et al., 1997). In addition to these genes, four more putative bacteriocins were identified and remain to be characterized. The species selectivity of these putative bacteriocins in SAG is currently being investigated.

Interspecies SilCR induction of SilA-responsive genes has been described previously between GAS and GGS (Belotserkovsky et al., 2009). Inter- and intra- species competition in streptococci can occur in a polymicrobial environment such as the oral cavity. The hypothetical mature SilCR_SAG-A_ and SilCR_SAG-B_ peptides are 26 and 25 amino acids respectively, while the mature GAS peptide is 17. Both SAG immature peptides have the same six amino acids at their 5′ end but vary at their 3′ end. The SAG SilCR peptides are not very similar to the GAS and GGS SilCR peptides in either sequence or length and it remains to be determined if these species’ *sil* systems are cross-reactive.

Our data demonstrates that *S. intermedius* B196 can sense and respond to SilCR_SAG-B_, which can be carried by all three SAG species; however, a much higher concentration of the foreign peptide SilCR_SAG-B_ was required to rescue the phenotype in B196Δ*silCR* than the B196-native version (SilCR_SAG-A_). This implies a divergent co-evolution of the *silCR* and *silB* genes in the SAG-A clade, with selection for self-detection.

SilCR expression has been shown to attenuate virulence in a mouse model of necrotizing fasciitis (Hidalgo-Grass et al., 2002, 2004). However, SilA has also been shown to promote expression of virulence-associated genes including streptolysin S, iron transporter SiaA and serine protease ScpC (Salim et al., 2008). It is unknown whether the *sil* system in SAG affects its pathogenicity. This system is unusual in that it can contribute to both infection in the host and bacterial competition depending on the environment. Given the host-specificity of SAG, and the commensal and pathogenic roles it can play, further analysis of this system could deepen our understanding of SAG competition and its associated clinical diseases.

## Author Contributions

All authors listed have made substantial, direct and intellectual contribution to the work and approved it for publication.

## Conflict of Interest Statement

The authors declare that the research was conducted in the absence of any commercial or financial relationships that could be construed as a potential conflict of interest.

## References

[B1] Altekar G. Dwarkadas S. Huelsenbeck J. P. Ronquist F. (2004). Parallel Metropolis coupled Markov chain Monte Carlo for Bayesian phylogenetic inference. *Bioinformatics* 20 407–415. 10.1093/bioinformatics/btg427 14960467

[B2] Altschul S. F. Madden T. L. Schäffer A. A. Zhang J. Zhang Z. Miller W. (1997). Gapped BLAST and PSI-BLAST: a new generation of protein database search programs. *Nucleic Acids Res.* 25 3389–3402. 10.1093/nar/25.17.3389 9254694 PMC146917

[B3] Andrews S. (2010). *FastQC: A Quality Control Tool for High Throughput Sequence Data*. Available at: http://www.bioinformatics.babraham.ac.uk/projects/fastqc

[B4] Armstrong B. D. Herfst C. A. Tonial N. C. Wakabayashi A. T. Zeppa J. J. McCormick J. K. (2016). Identification of a two-component Class IIb bacteriocin in *Streptococcus pyogenes* by recombinase-based in vivo expression technology. *Sci. Rep.* 6:636233 10.1038/srep36233 PMC5093712 27808235

[B5] Asam D. Spellerberg B. (2014). Molecular pathogenicity of *Streptococcus anginosus*. *Mol. Oral. Microbiol.* 29 145–155. 10.1111/omi.12056 24848553

[B6] Bankevich A. Nurk S. Antipov D. Gurevich A. A. Dvorkin M. Kulikov A. S. (2012). SPAdes: a new genome assembly algorithm and its applications to single-cell sequencing. *J. Comput. Biol.* 19 455–477. 10.1089/cmb.2012.0021 22506599 PMC3342519

[B7] Belotserkovsky I. Baruch M. Peer A. Dov E. Ravins M. Mishalian I. (2009). Functional analysis of the quorum-sensing streptococcal invasion locus (sil). *PLoS Pathog.* 5:e1000651 10.1371/journal.ppat.1000651 PMC2766830 19893632

[B8] Benson D. A. Clark K. Karsch-Mizrachi I. Lipman D. J. Ostell J. Sayers E. W. (2015). GenBank. *Nucleic Acids Res.* 43 D30–D35. 10.1093/nar/gku1216 25414350 PMC4383990

[B9] Blin K. Kazempour D. Wohlleben W. Weber T. (2014). Improved lanthipeptide detection and prediction for antiSMASH. *PLoS ONE* 9:e89420 10.1371/journal.pone.0089420 PMC3930743 24586765

[B10] Blin K. Medema M. H. Kazempour D. Fischbach M. A. Breitling R. Takano E. (2013). antiSMASH 2.0–a versatile platform for genome mining of secondary metabolite producers. *Nucleic Acids Res.* 41 W204–W212. 10.1093/nar/gkt449 23737449 PMC3692088

[B11] Boetzer M. Henkel C. V. Jansen H. J. Butler D. Pirovano W. (2011). Scaffolding pre-assembled contigs using SSPACE. *Bioinformatics* 27 578–579. 10.1093/bioinformatics/btq683 21149342

[B12] Chikhi R. Medvedev P. (2014). Informed and automated k-mer size selection for genome assembly. *Bioinformatics* 30 31–37. 10.1093/bioinformatics/btt310 23732276

[B13] Cock P. J. A. Antao T. Chang J. T. Chapman B. A. Cox C. J. Dalke A. (2009). Biopython: freely available Python tools for computational molecular biology and bioinformatics. *Bioinformatics* 25 1422–1423. 10.1093/bioinformatics/btp163 19304878 PMC2682512

[B14] Coman G. Pânzaru C. Diculencu D. Gotia D. Cârlan M. Dahorea C. (1995). Pyogenic infections with different locations caused by *Streptococcus anginosus* alone or in association with anaerobic bacteria. *Rev. Med. Chir. Soc. Med. Nat. Iasi* 99 215–219. 9455370

[B15] Crooks G. E. Hon G. Chandonia J.-M. Brenner S. E. (2004). WebLogo: a sequence logo generator. *Genome Res.* 14 1188–1190. 10.1101/gr.849004 15173120 PMC419797

[B16] Dawid S. Roche A. M. Weiser J. N. (2007). The blp bacteriocins of *Streptococcus pneumoniae* mediate intraspecies competition both in vitro and in vivo. *Infect. Immun.* 75 443–451. 10.1128/IAI.01775-05 17074857 PMC1828380

[B17] Delcher A. Harmon D. Kasif S. White O. Salzberg S. L. (1999). Improved microbial gene identification with GLIMMER. *Nucleic Acids Res.* 27 4636–4641. 10.1093/nar/27.23.4636 10556321 PMC148753

[B18] Dunny G. M. Lee L. N. LeBlanc D. J. (1991). Improved electroporation and cloning vector system for gram-positive bacteria. *Appl. Environ. Microbiol.* 57 1194–1201. 1905518 10.1128/aem.57.4.1194-1201.1991 PMC182867

[B19] Edgar R. C. (2004). MUSCLE: multiple sequence alignment with high accuracy and high throughput. *Nucleic Acids Res.* 32 1792–1797. 10.1093/nar/gkh340 15034147 PMC390337

[B20] Eran Y. Getter Y. Baruch M. Belotserkovsky I. Padalon G. Mishalian I. (2007). Transcriptional regulation of the sil locus by the SilCR signalling peptide and its implications on group A streptococcus virulence. *Mol. Microbiol.* 63 1209–1222. 10.1111/j.1365-2958.2007.05581.x 17238919

[B21] Fei F. Mendonca M. L. McCarry B. E. Bowdish D. M. E. Surette M. G. (2016). Metabolic and transcriptomic profiling of *Streptococcus intermedius* during aerobic and anaerobic growth. *Metabolomics* 12:46 10.1007/s11306-016-0966-0

[B22] Fischer S. Brunk B. P. Chen F. Gao X. Harb O. S. Iodice J. B. (2011). Using OrthoMCL to assign proteins to OrthoMCL-DB groups or to cluster proteomes into new ortholog groups. *Curr. Protoc. Bioinformatics* Chapter 6 1–19. 10.1002/0471250953.bi0612s35 PMC3196566 21901743

[B23] Goujon M. McWilliam H. Li W. Valentin F. Squizzato S. Paern J. (2010). A new bioinformatics analysis tools framework at EMBL-EBI. *Nucleic Acids Res.* 38 W695–W699. 10.1093/nar/gkq313 20439314 PMC2896090

[B24] Gurevich A. Saveliev V. Vyahhi N. Tesler G. (2013). QUAST: quality assessment tool for genome assemblies. *Bioinformatics* 29 1072–1075. 10.1093/bioinformatics/btt086 23422339 PMC3624806

[B25] Hammami R. Zouhir A. Ben Hamida J. Fliss I. Jack R. Tagg J. (2007). BACTIBASE: a new web-accessible database for bacteriocin characterization. *BMC Microbiol.* 7:89 10.1186/1471-2180-7-89 PMC2211298 17941971

[B26] Hammami R. Zouhir A. Le Lay C. Ben Hamida J. Fliss I. Riley M. (2010). BACTIBASE second release: a database and tool platform for bacteriocin characterization. *BMC Microbiol.* 10:22 10.1186/1471-2180-10-22 PMC2824694 20105292

[B27] Hidalgo-Grass C. Dan-Goor M. Maly A. Eran Y. Kwinn L. A. Nizet V. (2004). Effect of a bacterial pheromone peptide on host chemokine degradation in group A streptococcal necrotising soft-tissue infections. *Lancet* 363 696–703. 10.1016/S0140-6736(04)15643-2 15001327

[B28] Hidalgo-Grass C. Ravins M. Dan-Goor M. Jaffe J. Moses A. E. Hanski E. (2002). A locus of group A *Streptococcus* involved in invasive disease and DNA transfer. *Mol. Microbiol.* 46 87–99. 10.1046/j.1365-2958.2002.03127.x 12366833

[B29] Huelsenbeck J. P. Ronquist F. (2001). MRBAYES: Bayesian inference of phylogenetic trees. *Bioinformatics* 17 754–755. 10.1093/bioinformatics/17.8.754 11524383

[B30] Jacobs J. A. Pietersen H. G. Stobberingh E. E. Soeters P. B. (1995). *Streptococcus anginosus, Streptococcus constellatus* and *Streptococcus intermedius*. Clinical relevance, hemolytic and serologic characteristics. *Am. J. Clin. Pathol.* 104 547–553. 10.1093/ajcp/104.5.547 7572815

[B31] Jensen A. Hoshino T. Kilian M. (2013). Taxonomy of the Anginosus group of the genus *Streptococcus* and description of *Streptococcus anginosus* subsp. whileyi subsp. nov. and *Streptococcus constellatus* subsp. viborgensis subsp. nov. *Int. J. Syst. Evol. Microbiol.* 63 2506–2519. 10.1099/ijs.0.043232-0 23223817

[B32] Jimenez J. C. Federle M. J. (2014). Quorum sensing in group A *Streptococcus*. *Front. Cell Infect. Microbiol.* 4:127 10.3389/fcimb.2014.00127 PMC4162386 25309879

[B33] Kaiser J. C. Verschoor C. P. Surette M. G. Bowdish D. M. (2014). Host cytokine responses distinguish invasive from airway isolates of the *Streptococcus* milleri/anginosis group. *BMC Infect. Dis.* 14:498 10.1186/1471-2334-14-498 PMC4175566 25209732

[B34] Kearse M. Moir R. Wilson A. Stones-Havas S. Cheung M. Sturrock S. (2012). Geneious Basic: an integrated and extendable desktop software platform for the organization and analysis of sequence data. *Bioinformatics* 28 1647–1649. 10.1093/bioinformatics/bts199 22543367 PMC3371832

[B35] Kizy A. E. Neely M. N. (2009). First *Streptococcus pyogenes* signature-tagged mutagenesis screen identifies novel virulence determinants. *Infect. Immun.* 77 1854–1865. 10.1128/IAI.01306-08 19223485 PMC2681771

[B36] Kormin S. Rusul G. Radu S. Ling F. H. (2001). Bacteriocin-producing lactic Acid bacteria isolated from traditional fermented food. *Malays. J. Med. Sci. MJMS* 8 63–68. 22973159 PMC3433967

[B37] Laupland K. B. Ross T. Church D. L. Gregson D. B. (2006). Population-based surveillance of invasive pyogenic streptococcal infection in a large Canadian region. *Clin. Microbiol. Infect.* 12 224–230. 10.1111/j.1469-0691.2005.01345.x 16451408

[B38] Letunic I. Bork P. (2016). Interactive tree of life (iTOL) v3: an online tool for the display and annotation of phylogenetic and other trees. *Nucleic Acids Res.* 44 W242–W245. 10.1093/nar/gkw290 27095192 PMC4987883

[B39] Lewis P. O. (2001). A likelihood approach to estimating phylogeny from discrete morphological character data. *Syst. Biol.* 50 913–925. 10.1080/106351501753462876 12116640

[B40] Marciset O. Jeronimus-Stratingh M. C. Mollet B. Poolman B. (1997). Thermophilin 13, a nontypical antilisterial poration complex bacteriocin, that functions without a receptor. *J. Biol. Chem.* 272 14277–14284. 10.1074/jbc.272.22.14277 9162062

[B41] Maricic N. Dawid S. (2014). Using the overlay assay to qualitatively measure bacterial production of and sensitivity to pneumococcal bacteriocins. *J. Vis. Exp.* 91:e51876 10.3791/51876 PMC4235403 25350516

[B42] Martin M. (2011). Cutadapt removes adapter sequences from high-throughput sequencing reads. *EMBnet J.* 17:10 10.14806/ej.17.1.200

[B43] Michael-Gayego A. Dan-Goor M. Jaffe J. Hidalgo-Grass C. Moses A. E. (2013). Characterization of sil in invasive group A and G Streptococci: antibodies against bacterial pheromone peptide SilCR result in severe infection. *Infect. Immun.* 81 4121–4127. 10.1128/IAI.00359-13 23980111 PMC3811807

[B44] Olson A. B. Kent H. Sibley C. D. Grinwis M. E. Mabon P. Ouellette C. (2013). Phylogenetic relationship and virulence inference of *Streptococcus anginosus* Group: curated annotation and whole-genome comparative analysis support distinct species designation. *BMC Genomics* 14:895 10.1186/1471-2164-14-895 PMC3897883 24341328

[B45] Parkins M. D. Sibley C. D. Surette M. G. Rabin H. R. (2008). The *Streptococcus* milleri group–an unrecognized cause of disease in cystic fibrosis: a case series and literature review. *Pediatr. Pulmonol.* 43 490–497. 10.1002/ppul.20809 18383109

[B46] Peng Y. Leung H. C. M. Yiu S. M. Chin F. Y. L. (2011). Meta-IDBA: a de Novo assembler for metagenomic data. *Bioinformatics* 27 i94–i101. 10.1093/bioinformatics/btr216 21685107 PMC3117360

[B47] Poole P. M. Wilson G. (1979). Occurrence and cultural features of *Streptococcus* milleri in various body sites. *J. Clin. Pathol.* 32 764–768. 10.1136/jcp.32.8.764 512036 PMC1145805

[B48] Price M. N. Dehal P. S. Arkin A. P. (2009). FastTree: computing large minimum evolution trees with profiles instead of a distance matrix. *Mol. Biol. Evol.* 26 1641–1650. 10.1093/molbev/msp077 19377059 PMC2693737

[B49] Ripley R. T. Cothren C. C. Moore E. E. Long J. Johnson J. L. Haenel J. B. (2006). *Streptococcus* milleri infections of the pleural space: operative management predominates. *Am. J. Surg.* 192 817–821. 10.1016/j.amjsurg.2006.08.050 17161100

[B50] Ruoff K. L. (1988). *Streptococcus anginosus* (&quot;*Streptococcus* milleri&quot): the unrecognized pathogen. *Clin. Microbiol. Rev.* 1 102–108. 10.1128/CMR.1.1.102 3060239 PMC358032

[B51] Salim K. Y. de Azavedo J. C. Bast D. J. Cvitkovitch D. G. (2008). Regulation of sagA, siaA and scpC by SilCR, a putative signaling peptide of *Streptococcus pyogenes*. *FEMS Microbiol. Lett.* 289 119–125. 10.1111/j.1574-6968.2008.01375.x 19016875

[B52] Shinzato T. Saito A. (1995). The *Streptococcus* milleri group as a cause of pulmonary infections. *Clin. Infect. Dis.* 21(Suppl. 3) S238–S243. 10.1093/clind/21.Supplement_3.S238 8749672

[B53] Sibley C. D. Grinwis M. E. Field T. R. Parkins M. D. Norgaard J. C. Gregson D. B. (2010). McKay agar enables routine quantification of the “*Streptococcus* milleri” group in cystic fibrosis patients. *J. Med. Microbiol.* 59 534–540. 10.1099/jmm.0.016592-0 20093379

[B54] Sibley C. D. Parkins M. D. Rabin H. R. Duan K. Norgaard J. C. Surette M. G. (2008). A polymicrobial perspective of pulmonary infections exposes an enigmatic pathogen in cystic fibrosis patients. *Proc. Natl. Acad. Sci. U.S.A.* 105 15070–15075. 10.1073/pnas.0804326105 18812504 PMC2567494

[B55] Siegman-Igra Y. Azmon Y. Schwartz D. (2012). Milleri group streptococcus–a stepchild in the viridans family. *Eur. J. Clin. Microbiol. Infect. Dis.* 31 2453–2459. 10.1007/s10096-012-1589-7 22391759

[B56] Simpson J. T. Durbin R. (2012). Efficient de novo assembly of large genomes using compressed data structures. *Genome Res.* 22 549–556. 10.1101/gr.126953.111 22156294 PMC3290790

[B57] Tavare S. (1986). Some probabilistic and statistical problems in the analysis of DNA sequences. *Lect. Math. Life Sci.* 17 57–86.

[B58] van Rossum G. Drake F. L. (2001). *Python Reference Manual.* Virginia: PythonLabs Available at: http://www.python.org

[B59] Whiley R. A. Beighton D. Winstanley T. G. Fraser H. Y. Hardie J. M. (1992). *Streptococcus intermedius, Streptococcus constellatus*, and *Streptococcus anginosus* (the *Streptococcus* milleri group): association with different body sites and clinical infections. *J. Clin. Microbiol.* 30 243–244. 1734062 10.1128/jcm.30.1.243-244.1992 PMC265033

[B60] Whitford M. F. McPherson M. A. Forster R. J. Teather R. M. (2001). Identification of bacteriocin-like inhibitors from rumen *Streptococcus* spp. and isolation and characterization of bovicin 255. *Appl. Environ. Microbiol.* 67 569–574. 10.1128/AEM.67.2.569-574.2001 11157218 PMC92622

[B61] Yang Z. (1994). Maximum likelihood phylogenetic estimation from DNA sequences with variable rates over sites: approximate methods. *J. Mol. Evol.* 39 306–314. 10.1007/BF00160154 7932792

[B62] Zerbino D. R. Birney E. (2008). Velvet: algorithms for de novo short read assembly using de Bruijn graphs. *Genome Res.* 18 821–829. 10.1101/gr.074492.107 18349386 PMC2336801

[B63] Zimin A. V. Marçais G. Puiu D. Roberts M. Salzberg S. L. Yorke J. A. (2013). The MaSuRCA genome assembler. *Bioinformatics* 29 2669–2677. 10.1093/bioinformatics/btt476 23990416 PMC3799473

