# Supplementary material for: The sil Locus in Streptococcus Anginosus Group: Interspecies Competition and a Hotspot of Genetic Diversity
Source: Front Microbiol. 2017 Jan 10;7:2156. doi: 10.3389/fmicb.2016.02156 (PMC5222867; doi:10.3389/fmicb.2016.02156)
Supplement: Supplementary file 1 [file Table_1.DOCX]

**Table S1: Strains used in the analysis of *sil* loci in *Streptococcus* Anginosus Group**

| Strain Name | Isolated from | *sil* Classification | Genome Accession number | Sil Region Accession number |
| --- | --- | --- | --- | --- |
| ***S. intermedius* C270** | Airway | Group A | CP003858 | KY315481 |
| ***S. intermedius* C1365** | Blood | Group A |  | KY315479 |
| ***S. intermedius* C1390** | Brain | Group A |  | KY315480 |
| ***S. intermedius* FO413 (AFX001000004)** | Dental Plaque | Group A | AFX001000004 | KY315440 |
| ***S. intermedius* B196** | Hip abscess | Group A | CP003857 | KY315459 |
| ***S. intermedius* C1369** | Blood | Group A |  | KY315460 |
| ***S. intermedius* C260** | Invasive | Group A |  | KY315471 |
| ***S. intermedius* C1377** | Blood | Group A |  | KY315470 |
| ***S. intermedius* M331** | Airway | Group A |  | KY315477 |
| ***S. intermedius* JTH08 (NC018073)** | N/A | Group A |  | KY315476 |
| ***S. intermedius* ATCC27335** | Invasive | Group A |  | KY315473 |
| ***S. intermedius* SK54 (AJKN01000015)** | invasive | Group A | AJKN01000015 | KY315472 |
| ***S. intermedius* C1374** | Blood | Group A |  | KY315475 |
| ***S. intermedius* M60R** | Exacerbation ^1^ | Group A |  | KY315461 |
| ***S. intermedius* FO395 (AFXN01000007)** | Dental plaque | Group D | AFXN01000007 | KY315447 |
| ***S. anginosus* M423** | Airway | Group A |  | KY315478 |
| ***S. anginosus* M569** | Airway | Group E |  | KY315446 |
| ***S. anginosus* C984** | Airway | Group E |  | KY315444 |
| ***S. anginosus* M410** | Airway | Group E |  | KY315445 |
| ***S. anginosus* C252** | Invasive | Group E |  | KY315443 |
| ***S. anginosus* FO211 (AECT01000012)** | Nasopharynx | Group E | AECT01000012 | KY315442 |
| ***S. anginosus* SK52 (AREF010000001)** | Throat | Group F | AREF010000001 | KY315441 |
| ***S. anginosus* C238** | Exacerbation^1^ | Group A | CP003861 | KY315458 |
| ***S. anginosus* subsp. *whileyi* MAS624 (AP013072)** | Sore throat | Group A | AP013072 | KY315457 |
| ***S. anginosus* subsp. *whileyi* CCUG39159 (AICP01000048)** | Sore throat | Group B | AICP01000048 | KY315456 |
| ***S. anginosus* C1051** | Blood | Group B | CP003860 | KY315465 |
| ***S. anginosus* 1_2_62CV (ADME01000005)** | Crohn’s disease | Group A | ADME01000005 | KY315455 |
| ***S. constellatus* M505** | Airway | Group D |  | KY315448 |
| ***S. constellatus* subsp. *constellatus* SK53 (AICQ01000033)** | Purulent pleurisy | Group A | AICQ01000033 | KY315463 |
| ***S. constellatus* subsp*. constellatus* ATCC27823** | Invasive | Group A |  | KY315464 |
| ***S. constellatus* M47** | Airway | Group C |  | KY315462 |
| ***S. constellatus* C1379** | Empyema | Group A |  | KY315469 |
| ***S. constellatus* C1392** | Brain | Group A |  | KY315468 |
| ***S. constellatus* C1367** | Blood | Group A |  | KY315474 |
| ***S. constellatus* C1384** | Empyema | Group A |  | KY315467 |
| ***S. constellatus* C1366** | Blood | Group A |  | KY315466 |
| ***S. constellatus* subsp. *pharyngis* SK1060 (AFUP01000001)** | throat | Group A | AFUP01000001 | KY315449 |
| ***S. constellatus* M193** | Exacerbation^1^ | Group A |  | KY315454 |
| ***S. constellatus* subsp*. pharyngis* C818** |  | Group A | CP003840 | KY315453 |
| ***S. constellatus* subsp. *pharyngis* C1050** | Invasive | Group A | CP003859 | KY315450 |
| ***S. constellatus* C188** |  | Group A |  | KY315451 |
| ***S. constellatus* subsp*. pharyngis* C232** | airway | Group A | CP003800 | KY315452 |

^1^ isolated from sputum during pulmonary exacerbation in cystic fibrosis patient
