# Supplementary material for: The sil Locus in Streptococcus Anginosus Group: Interspecies Competition and a Hotspot of Genetic Diversity
Source: Front Microbiol. 2017 Jan 10;7:2156. doi: 10.3389/fmicb.2016.02156 (PMC5222867; doi:10.3389/fmicb.2016.02156)
Supplement: Supplementary file 2 [file Image_1.PDF]

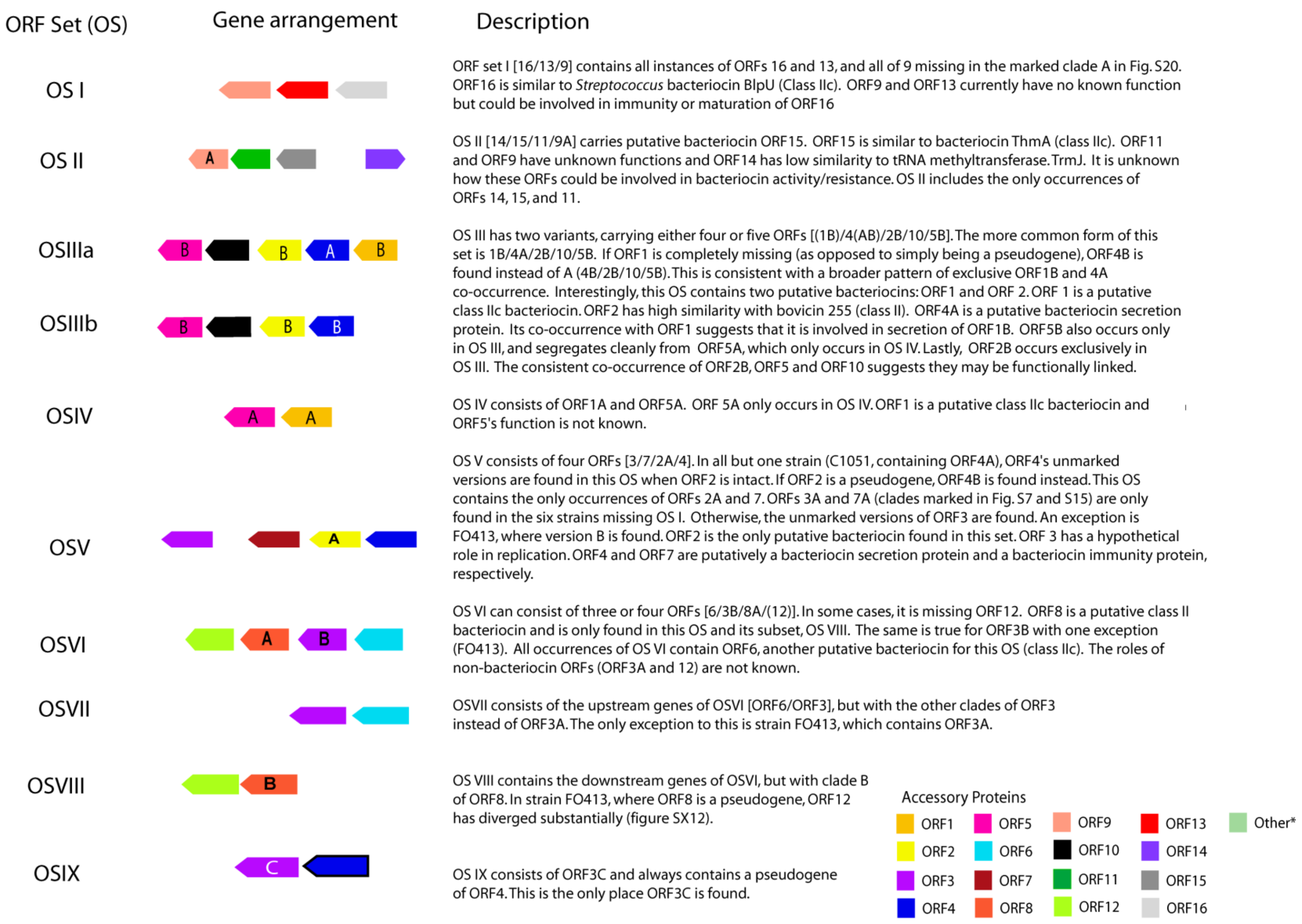

Figure S1: Organization of ORF sets (OS) within the bacteriocin accessory region based on synteny and co-occurrence. antiSMASH Glimmer was used to identify putative ORFs within the bacteriocin accessory region (Delcher et al. 1999; Blin et al. 2013). Sixteen ORFs classes were identified using orthoMCL, which were further divided into subclusters based on genetic distance (A-B labelling inside ORF).

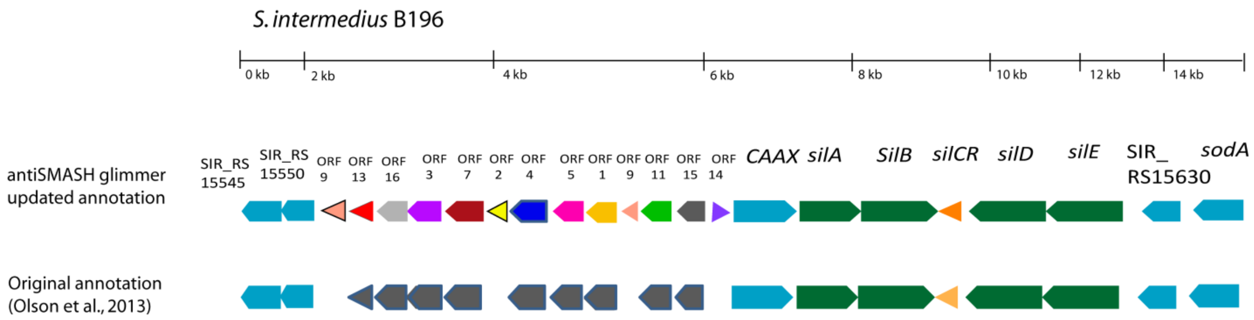

Figure S2: Comparison of annotations for bacteriocin accessory region in *Streptococcus intermedius* B196. antiSMASH glimmer was used to identify all putative ORFs within the accessory region after cross-referencing with other *Streptococcus* Anginosus Group genomes (Delcher et al. 1999; Blin et al. 2013; Blin et al. 2014). This is compared to the original annotation as published in Olson et al., 2013.

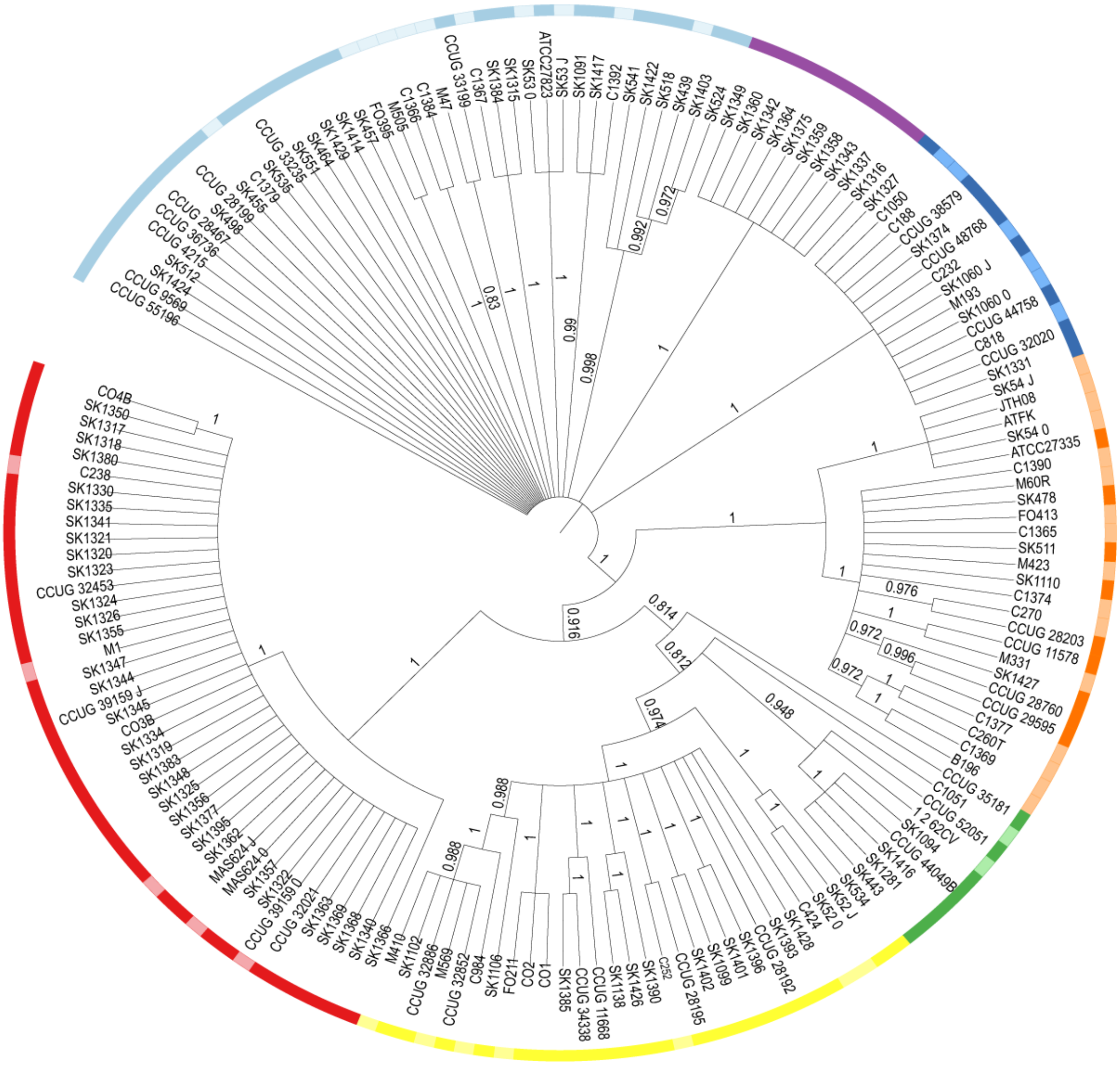

Figure S3: Multilocus sequence analysis (MLSA) tree of Streptococcus strains used including all strains from Jensen et al. 2013. Major clusters in our tree perfectly match those from Jensen et al. 2013, and are denoted using colours (cluster 1: orange, cluster 2: light blue, cluster 3: dark blue, cluster 4: purple, cluster 5: green, cluster 6: yellow, and cluster 7: red). Strains used in this study can be found nested in all clusters except cluster 4, and can be identified by their lighter colouring. Two strains (M423 and FO395) had incorrect species assignments according to the MLSA tree. Certain strains are listed twice indicating the sequences used in our study ("O") and in Jensen et al. 2013 ("J"). Bootstrap values are displayed. Nodes with <0.8 bootstrap support have been collapsed.

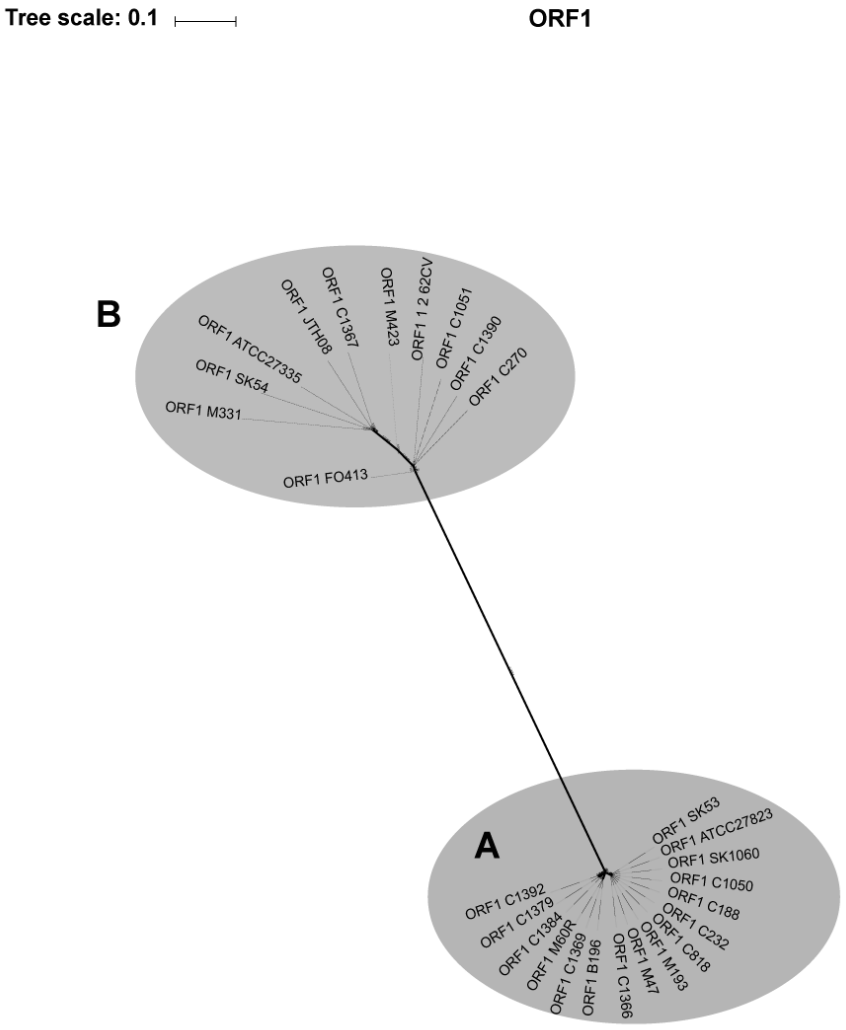

Figure S4: Phylogenetic tree of ORF 1 in the *Streptococcus* Anginosus Group (SAG). The tree was generated using FastTree (Price et al. 2009). SAG strains are labelled with descriptions available in Table 1.

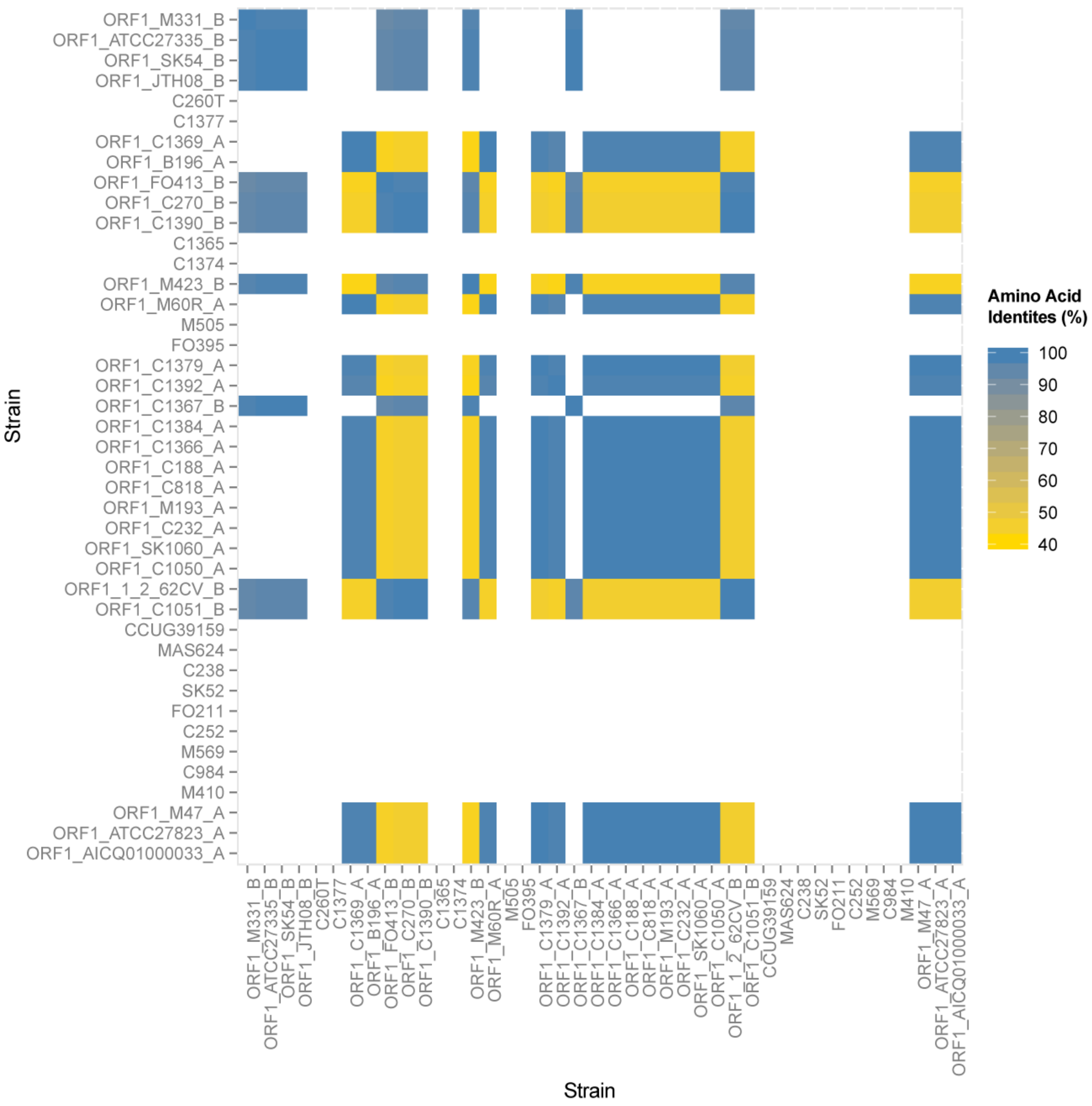

Figure S5: Amino acid identity of ORF 1 in *Streptococcus* Anginosus Group (SAG) strains. The strains are described in Table 1. Strains are ordered as shown in Figure 3.

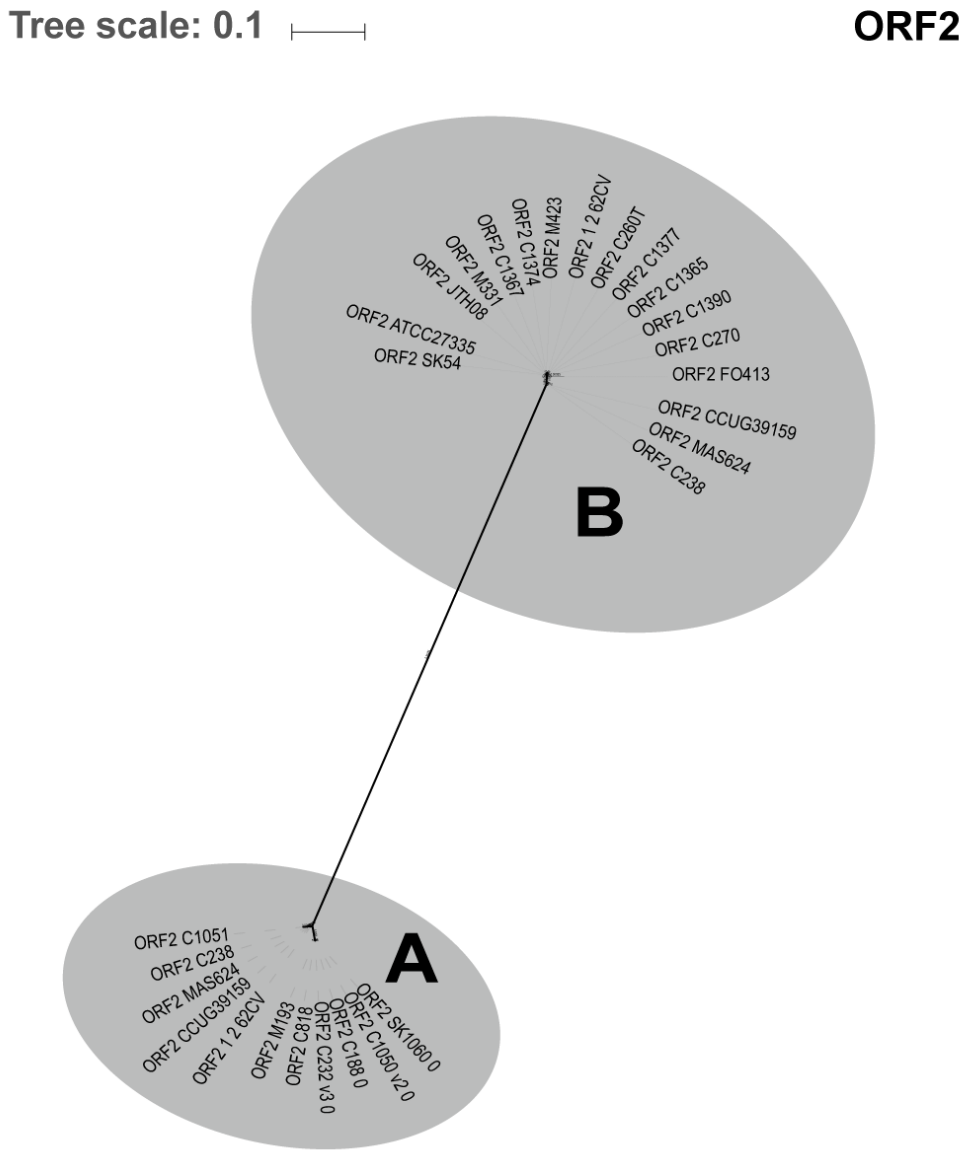

Figure S6: Phylogenetic tree ORF 2 in the *Streptococcus* Anginosus Group (SAG). The tree was generated using FastTree (Price et al. 2009). SAG strains are labelled with descriptions available in Table 1.

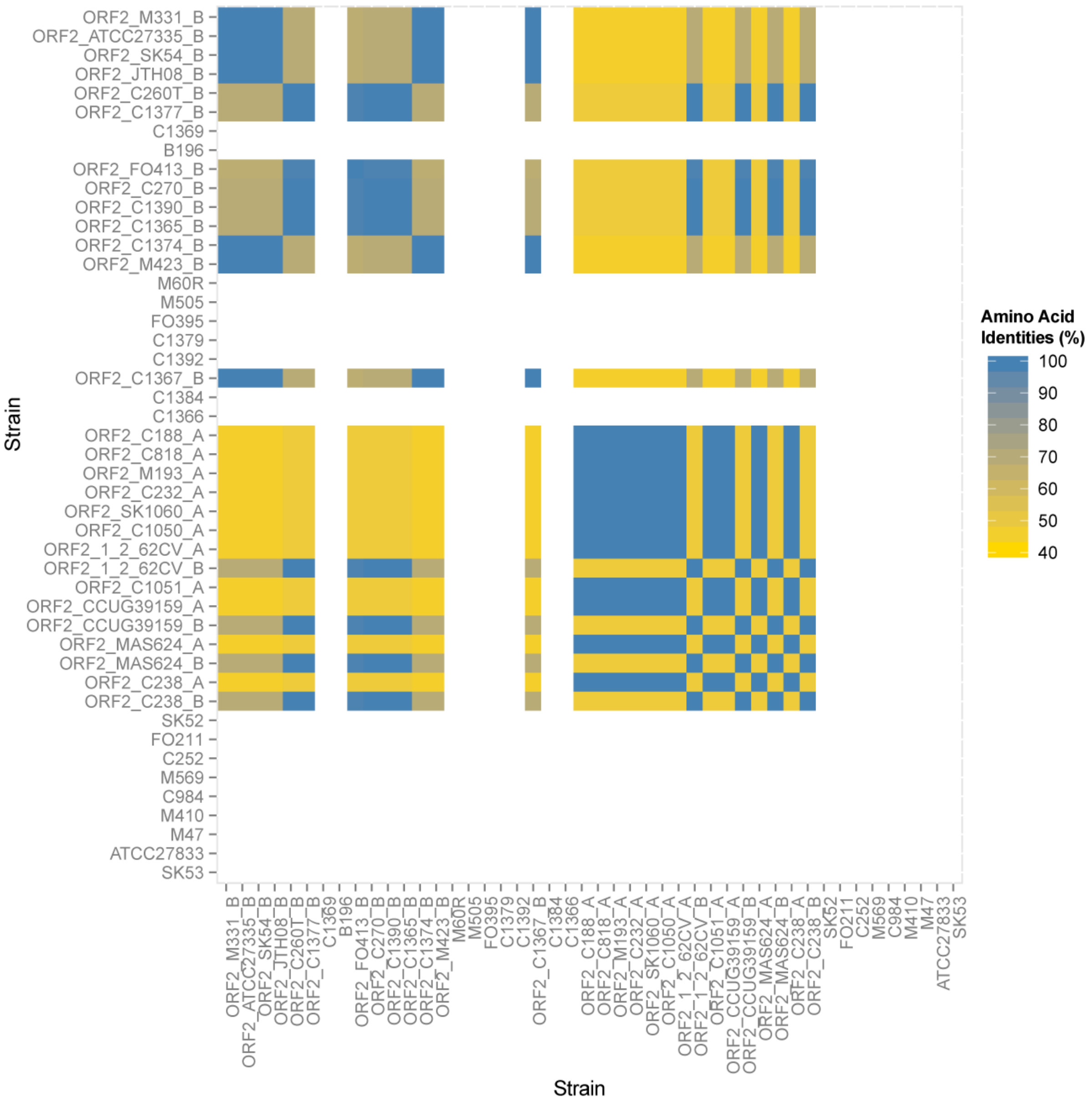

Figure S7: Amino acid identity of ORF 2 in *Streptococcus* Anginosus Group (SAG) strains. The strains are described in Table 1. Strains are ordered as shown in Figure 3.

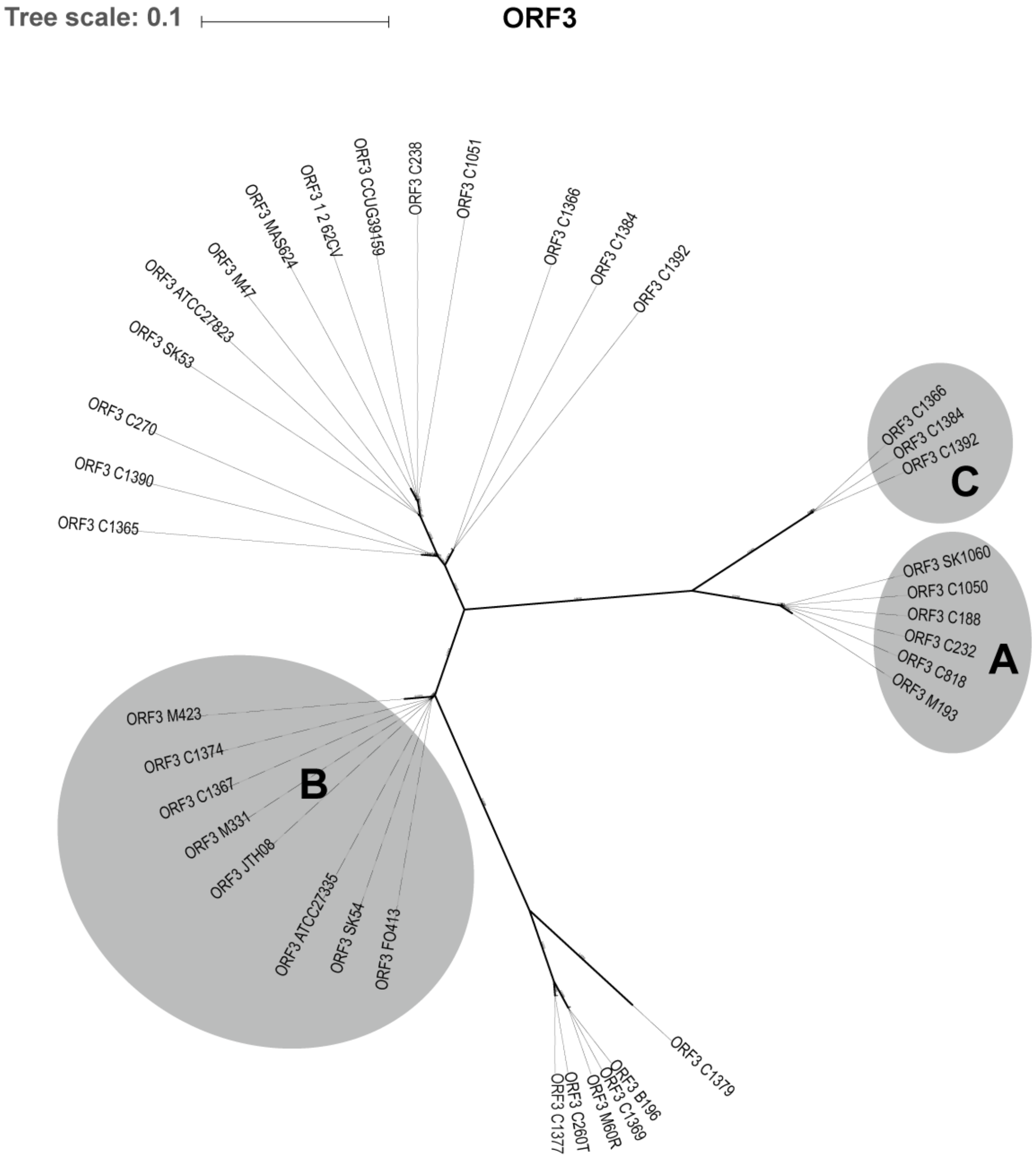

Figure S8: Phylogenetic tree of ORF 3 in the *Streptococcus* Anginosus Group (SAG). The tree was generated using FastTree (Price et al. 2009). SAG strains are labelled with descriptions available in Table 1.

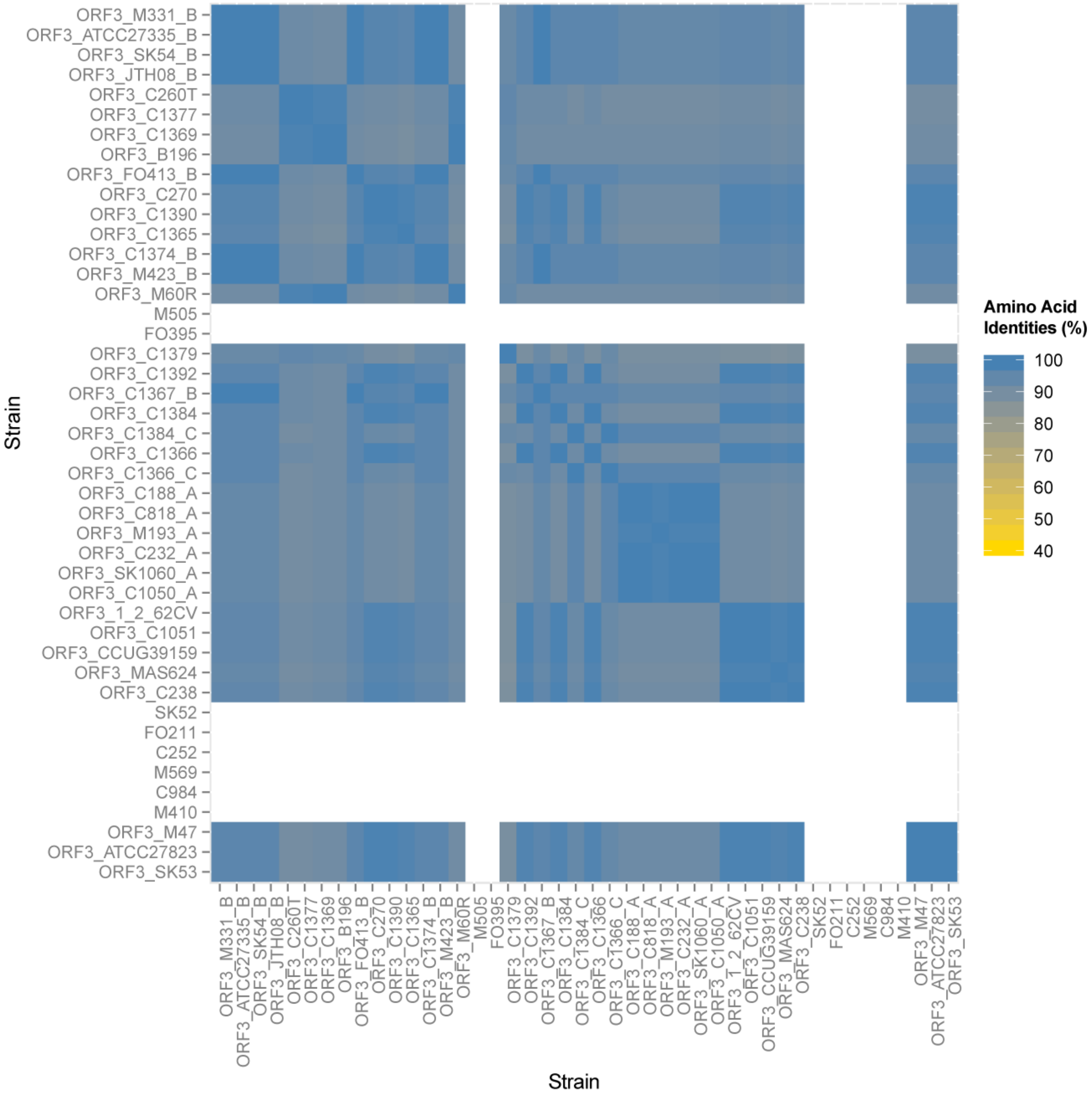

Fig S9: Amino acid identity of ORF 3 in *Streptococcus* Anginosus Group (SAG) strains. The strains are described in Table 1. Strains are ordered as shown in Figure 3.

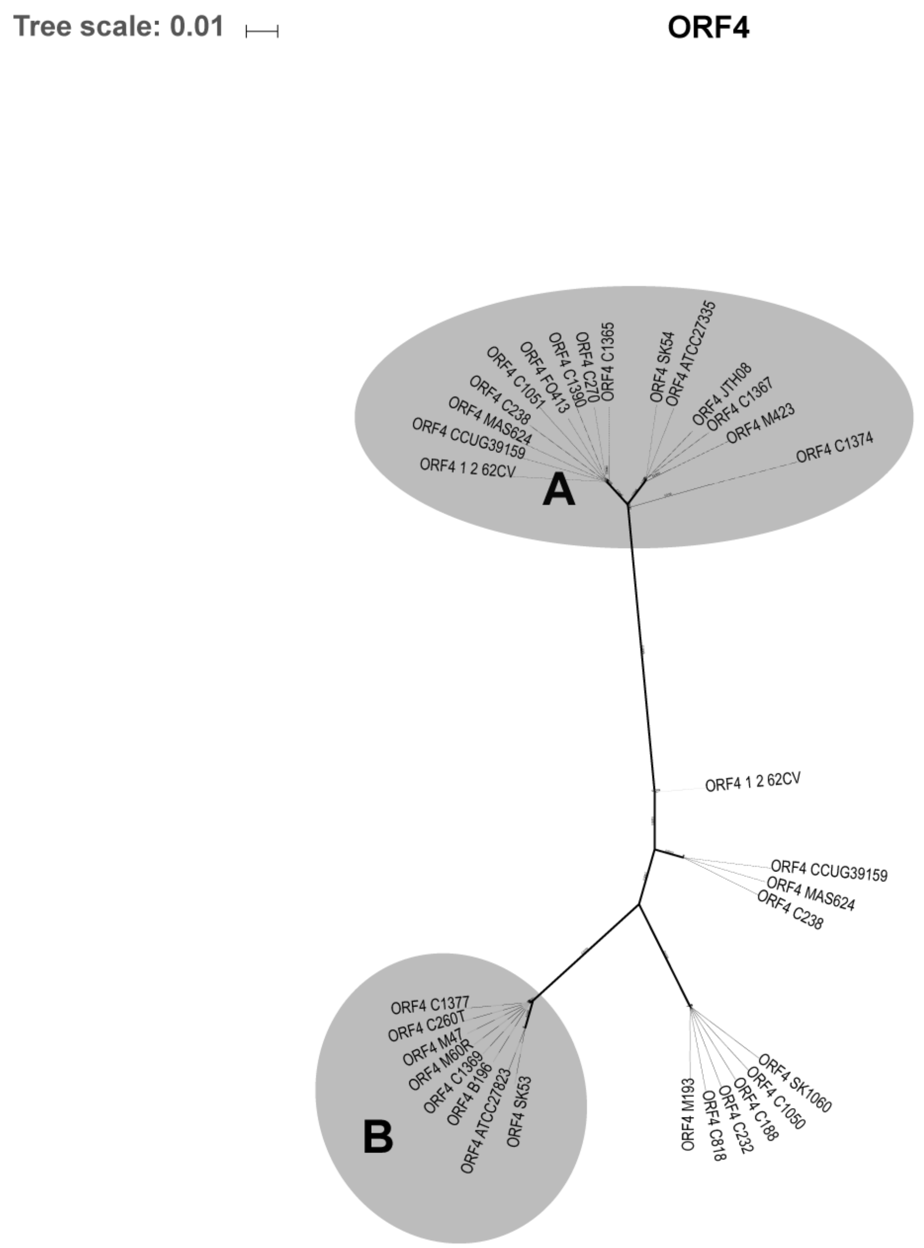

Figure S10: Phylogenetic tree of ORF 4 in the *Streptococcus* Anginosus Group (SAG). The tree was generated using FastTree (Price et al. 2009). SAG strains are labelled with descriptions available in Table 1.

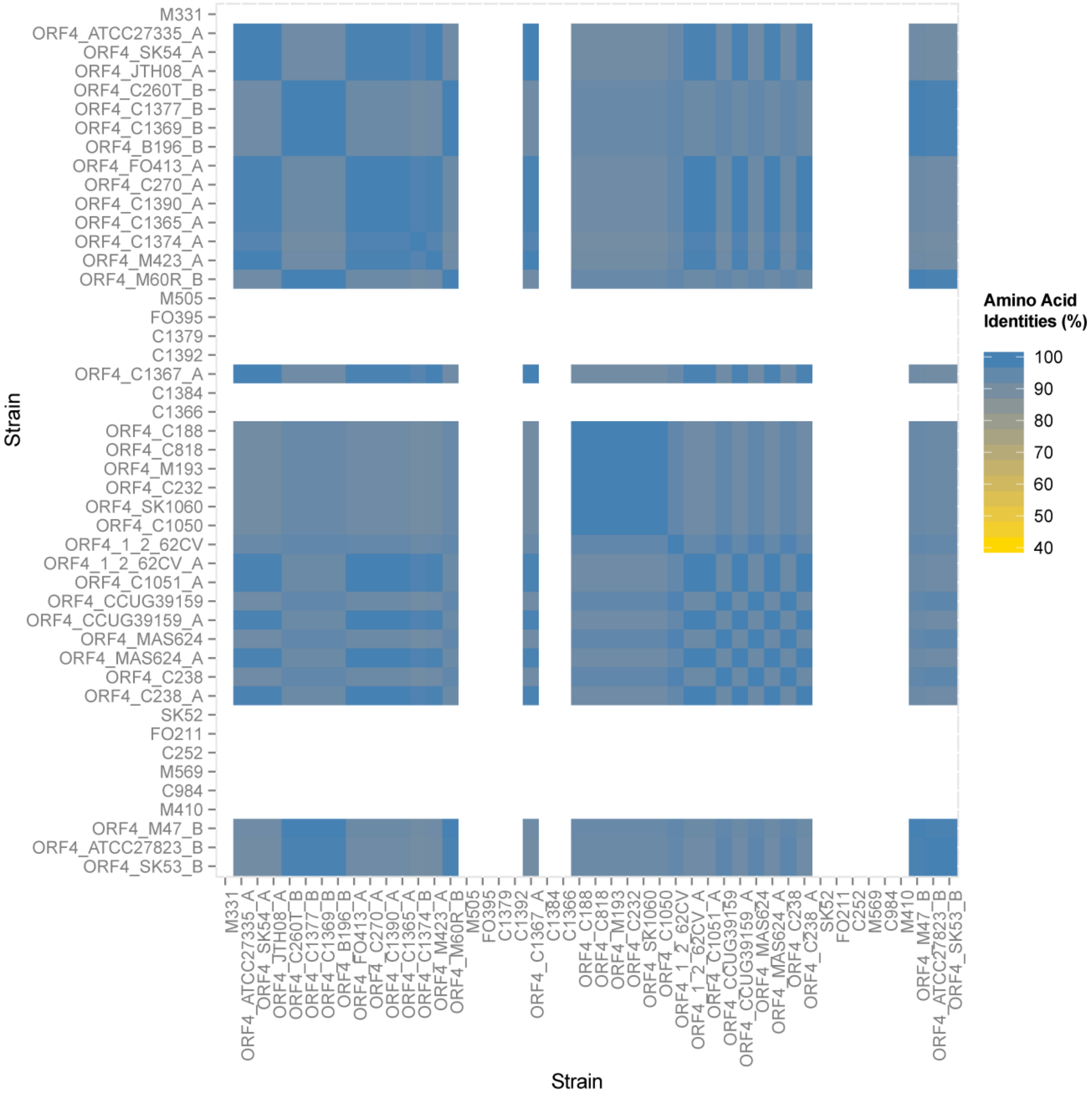

Figure S11: Amino acid identity of ORF 4 in *Streptococcus* Anginosus Group (SAG) strains. The strains are described in Table 1. Strains are ordered as shown in Figure 3.

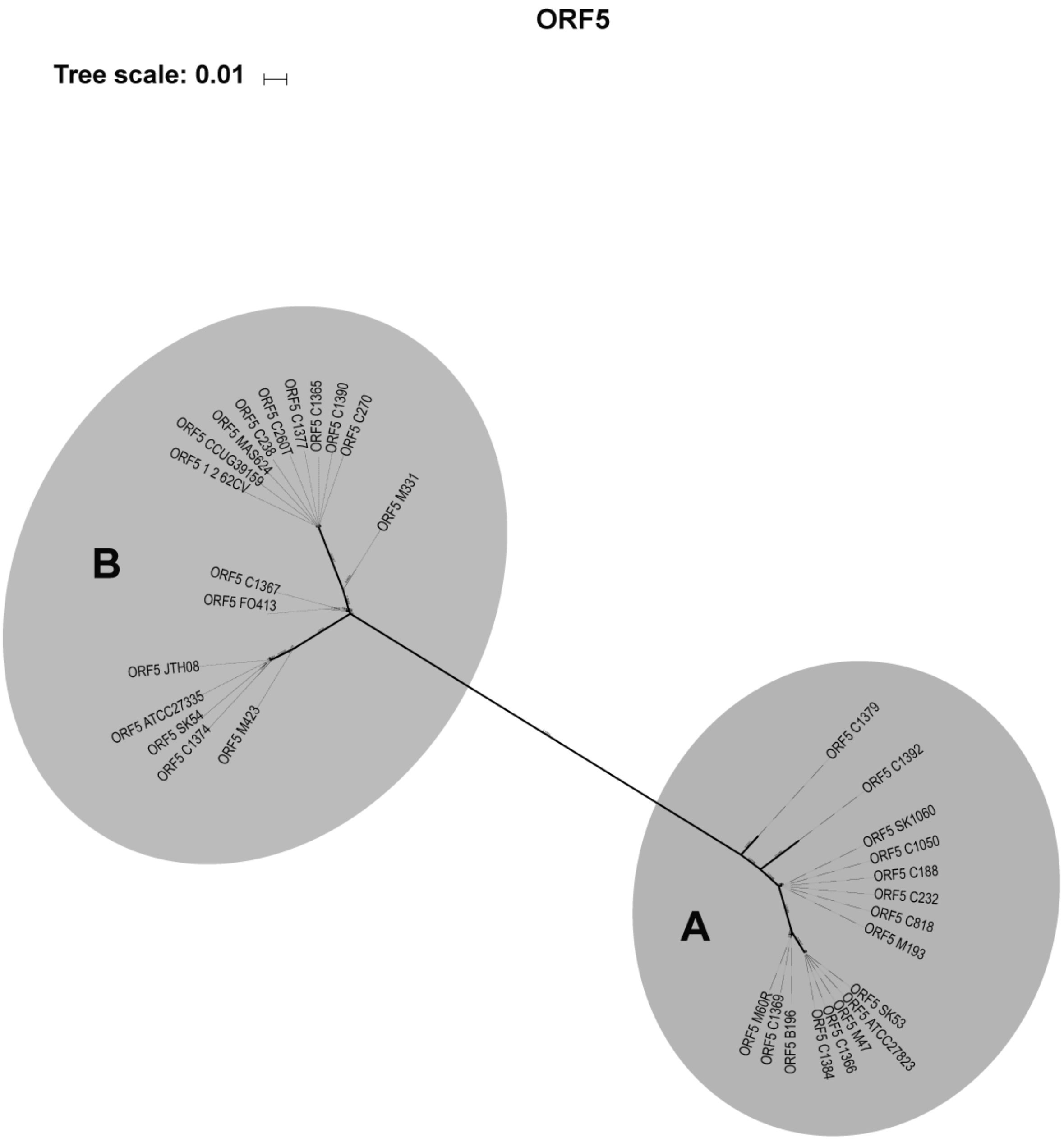

Figure S12: Phylogenetic tree of ORF 5 in the *Streptococcus* Anginosus Group (SAG). The tree was generated using FastTree (Price et al. 2009). SAG strains are labelled with descriptions available in Table 1.

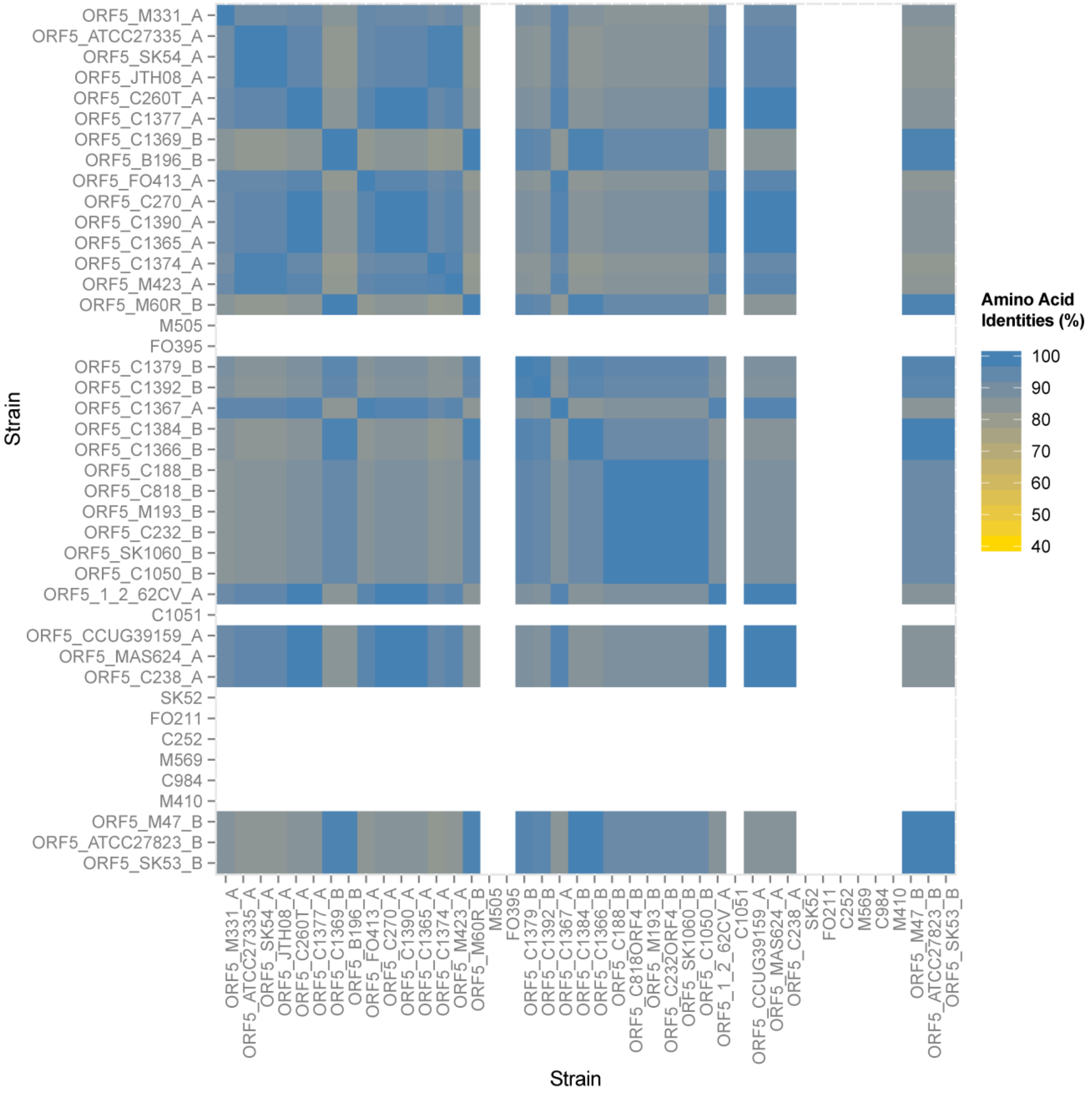

Figure S13: Amino acid identity of ORF 5 in *Streptococcus* Anginosus Group (SAG) strains. The strains are described in Table 1. Strains are ordered as shown in Figure 3.

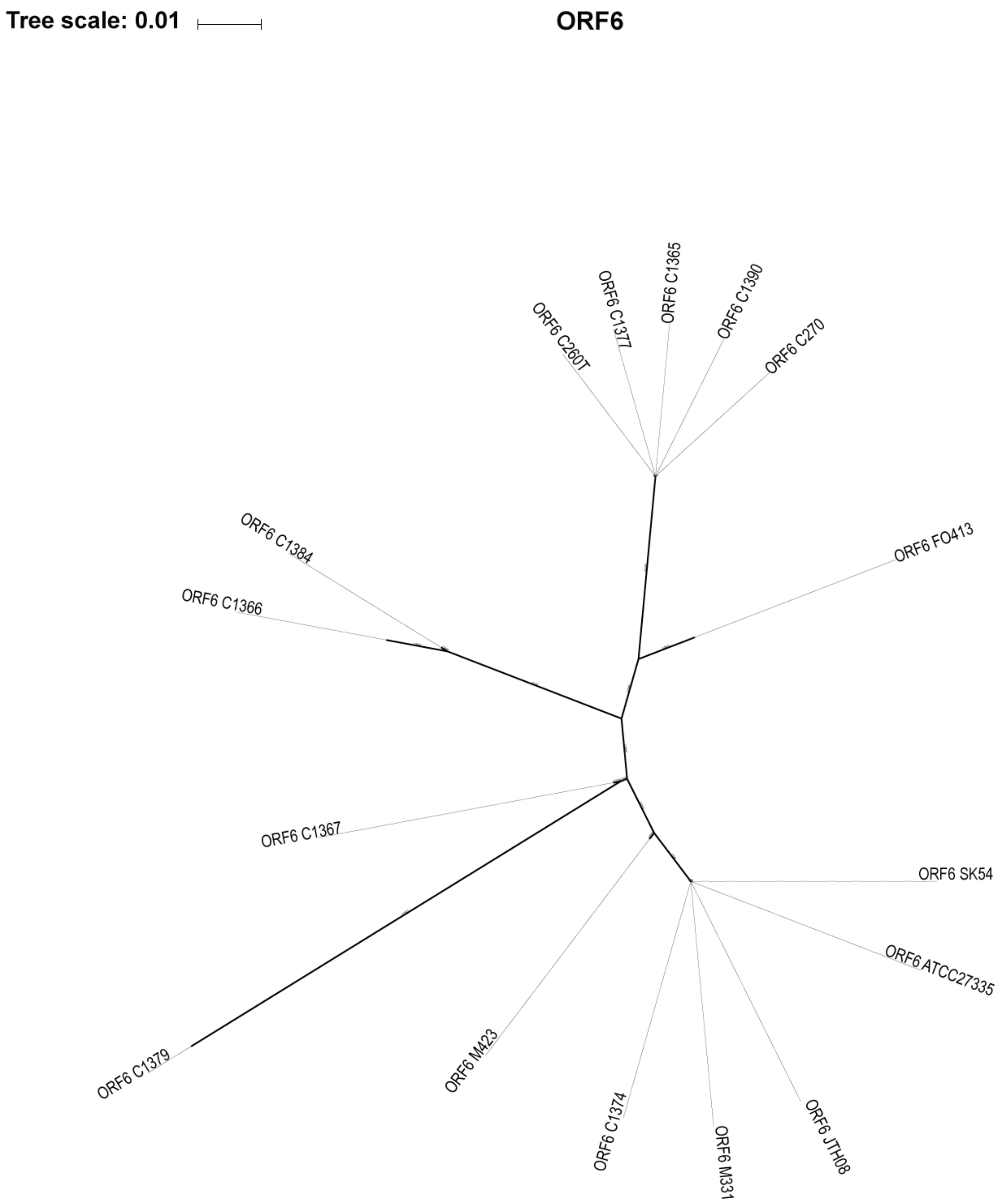

Figure S14: Phylogenetic tree of ORF 6 in the *Streptococcus* Anginosus Group (SAG). The tree was generated using FastTree (Price et al. 2009). SAG strains are labelled with descriptions available in Table 1.

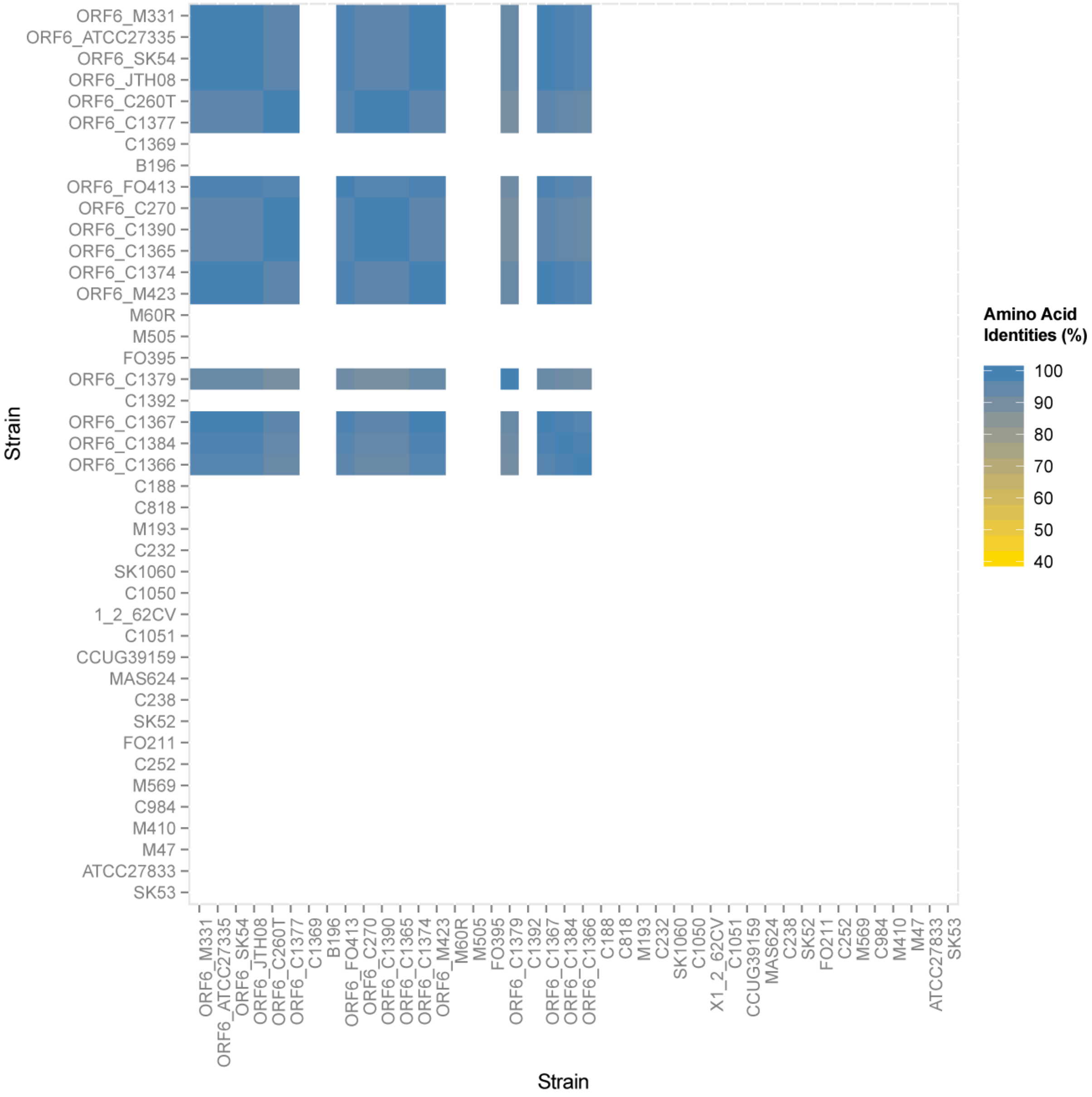

Figure S15: Amino acid identity of ORF 6 in *Streptococcus* Anginosus Group (SAG) strains. The strains are described in Table 1. Strains are ordered as shown in Figure 3.

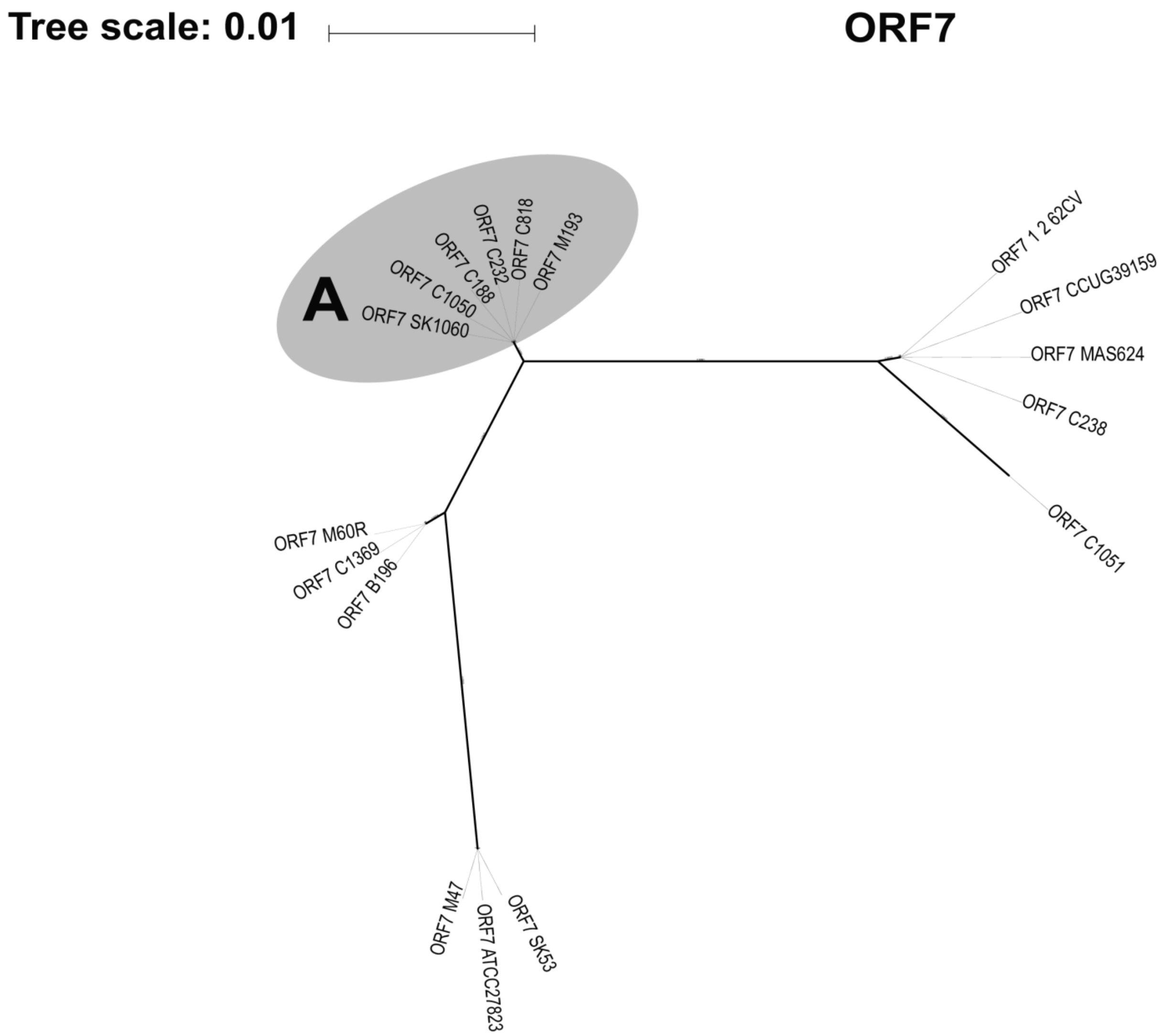

Figure S16: Phylogenetic tree of ORF 7 in the *Streptococcus* Anginosus Group (SAG). The tree was generated using FastTree (Price et al. 2009). SAG strains are labelled with descriptions available in Table 1.

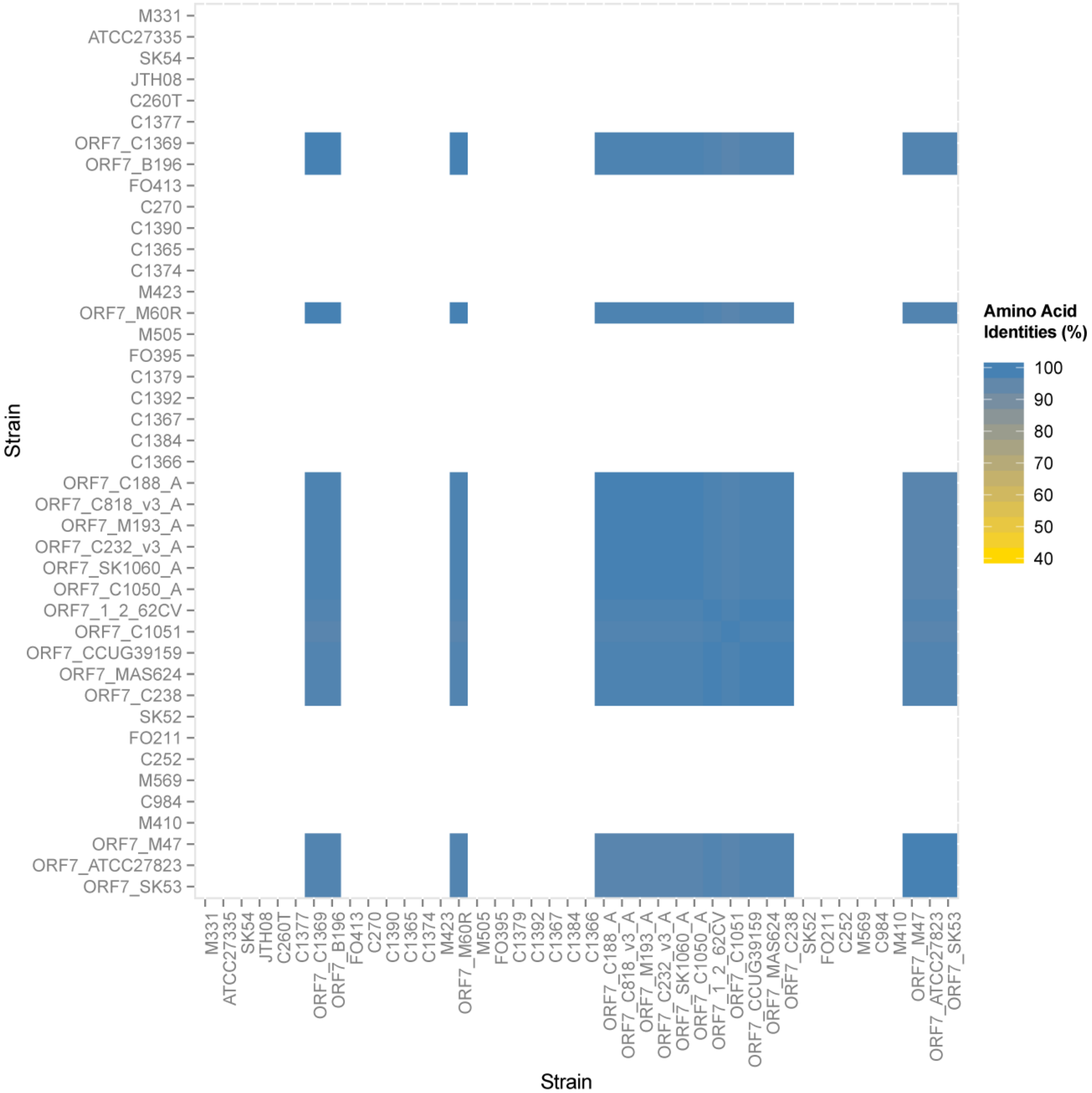

Figure S17: Amino acid identity of ORF 7 in *Streptococcus* Anginosus Group (SAG) strains. The strains are described in Table 1. Strains are ordered as shown in Figure 3.

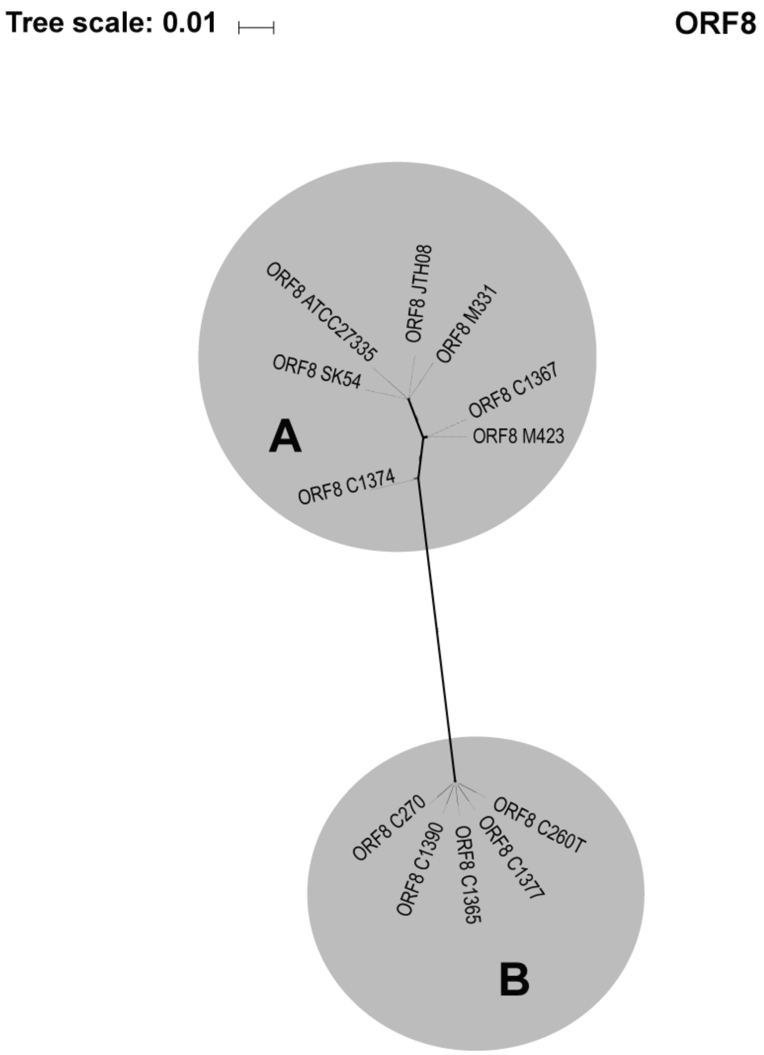

Figure S18: Phylogenetic tree of ORF 8 in the *Streptococcus* Anginosus Group (SAG). The tree was generated using FastTree (Price et al. 2009). SAG strains are labelled with descriptions available in Table 1.

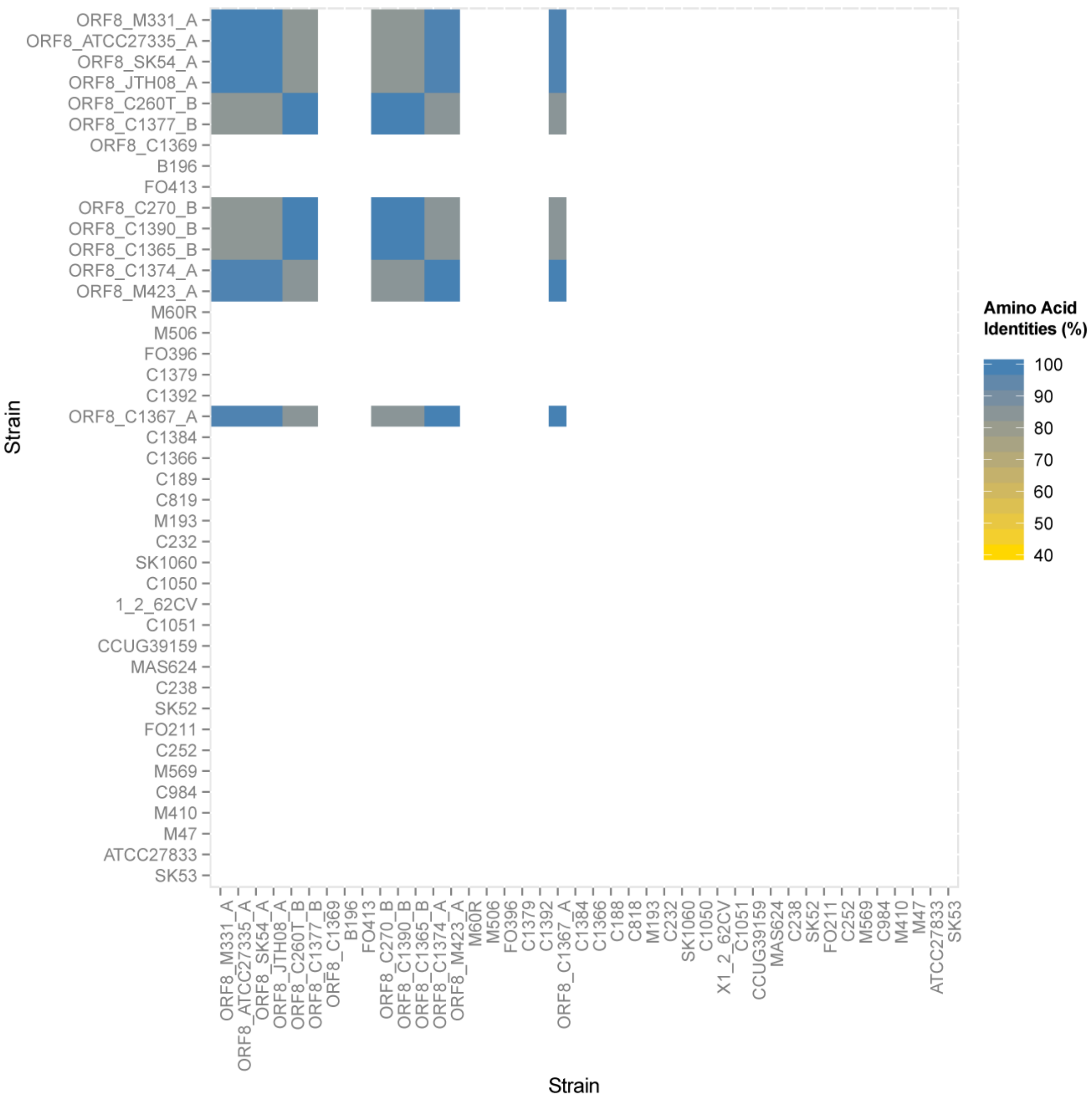

Figure S19: Amino acid identity of ORF 8 in *Streptococcus* Anginosus Group (SAG) strains. The strains are described in Table 1. Strains are ordered as shown in Figure 3.

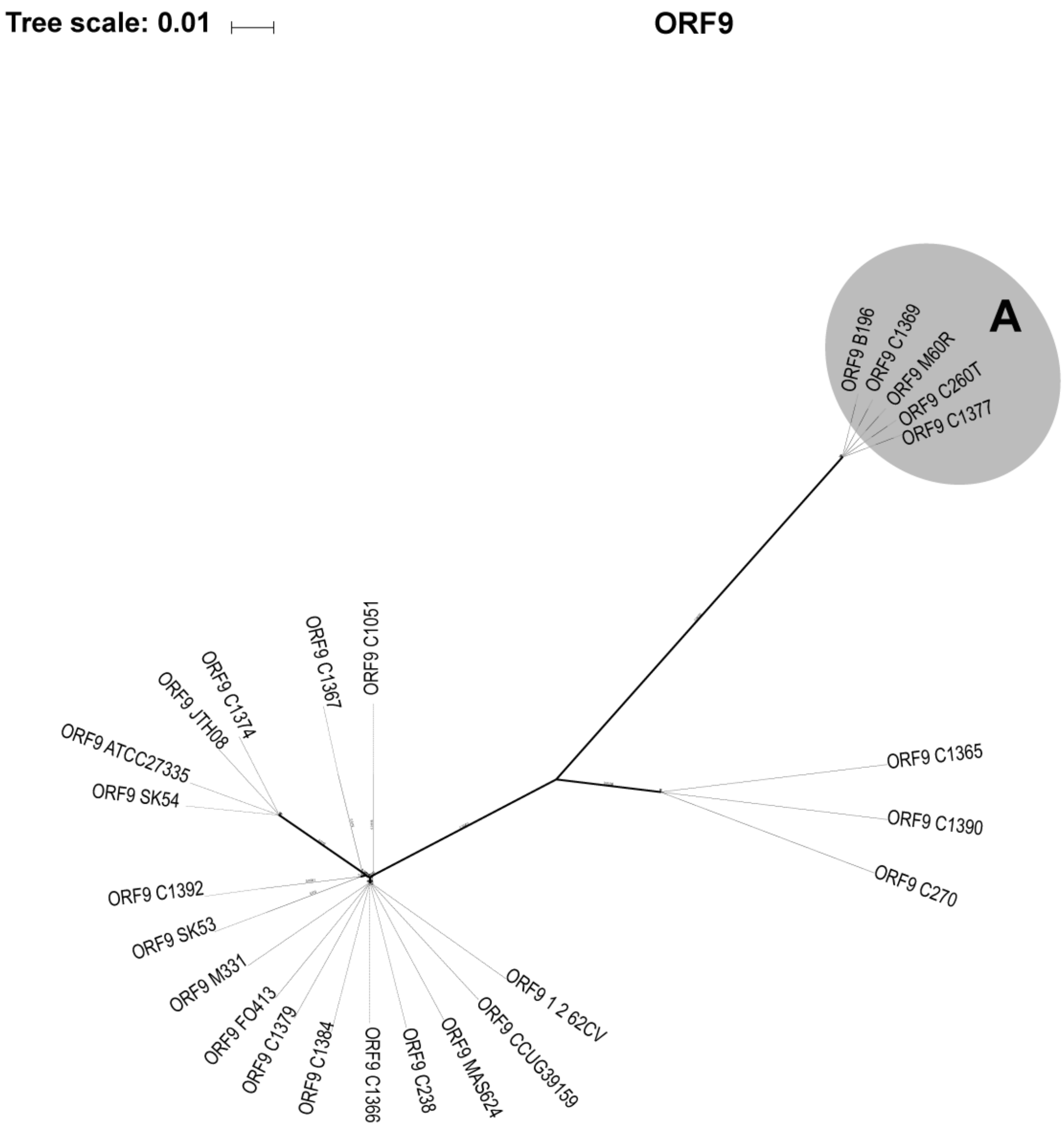

Figure S20: Phylogenetic tree of ORF 9 in the *Streptococcus* Anginosus Group (SAG). The tree was generated using FastTree (Price et al. 2009). SAG strains are labelled with descriptions available in Table 1.

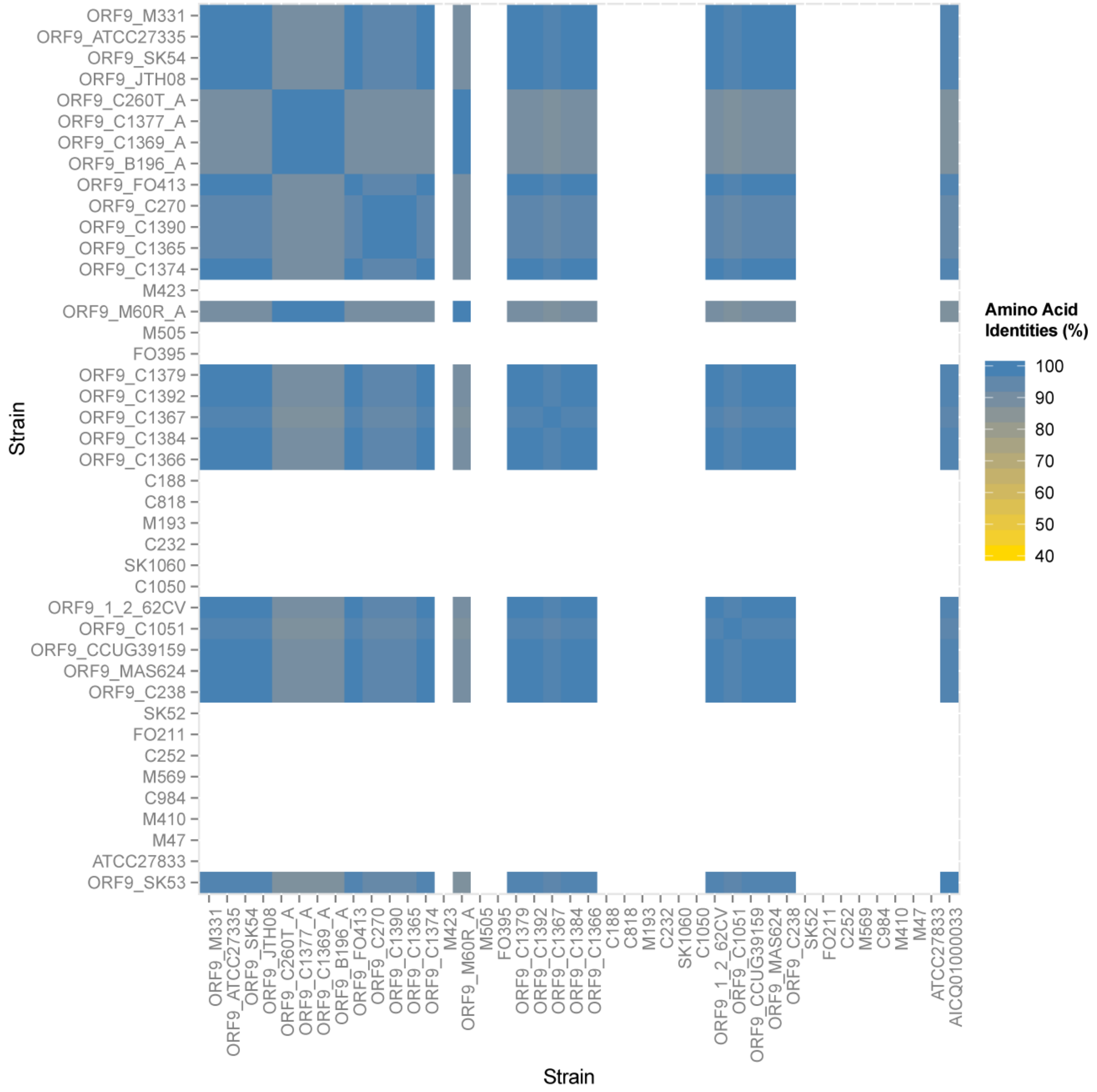

Figure S21: Amino acid identity of ORF 9 in *Streptococcus* Anginosus Group (SAG) strains. The strains are described in Table 1. Strains are ordered as shown in Figure 3.

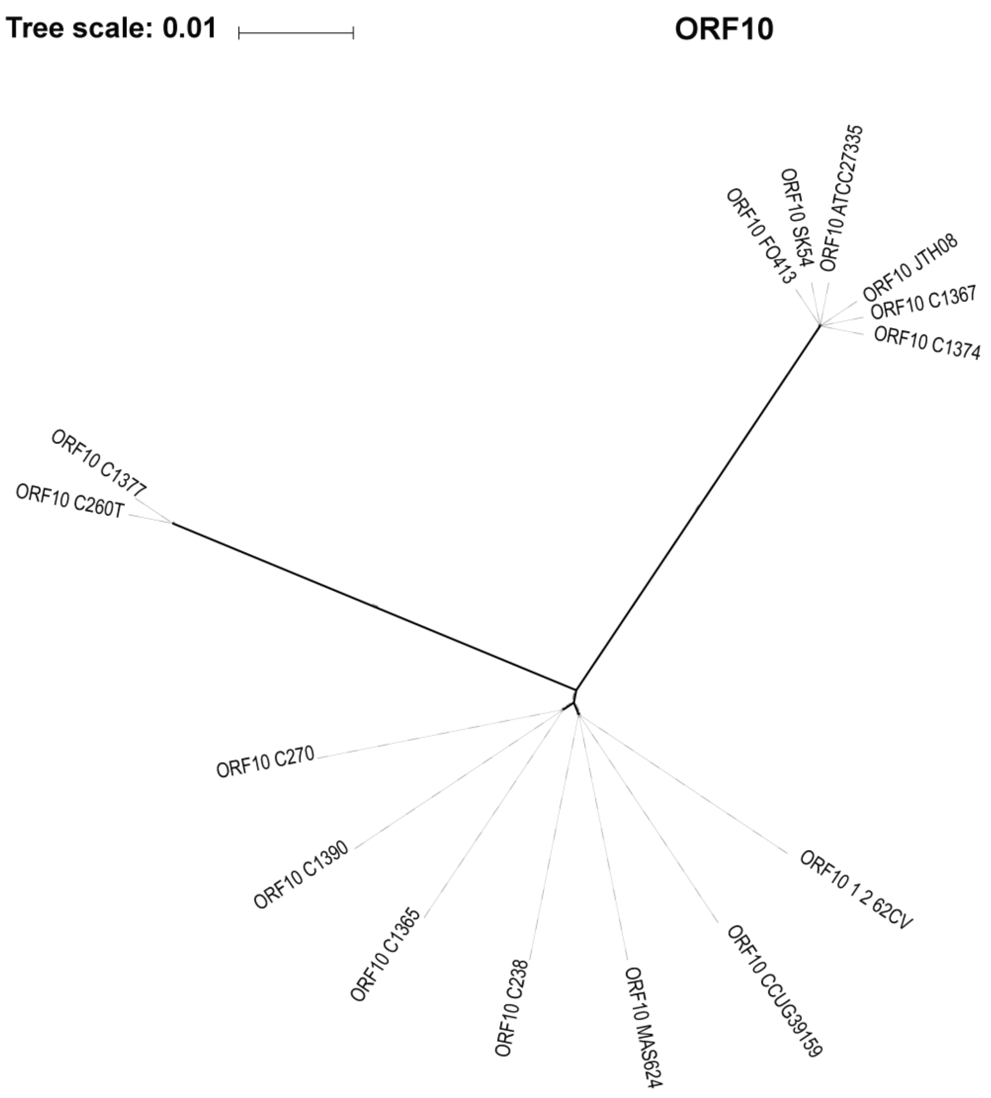

Figure S22: Phylogenetic tree of ORF 10 in the *Streptococcus* Anginosus Group (SAG). The tree was generated using FastTree (Price et al. 2009). SAG strains are labelled with descriptions available in Table 1.

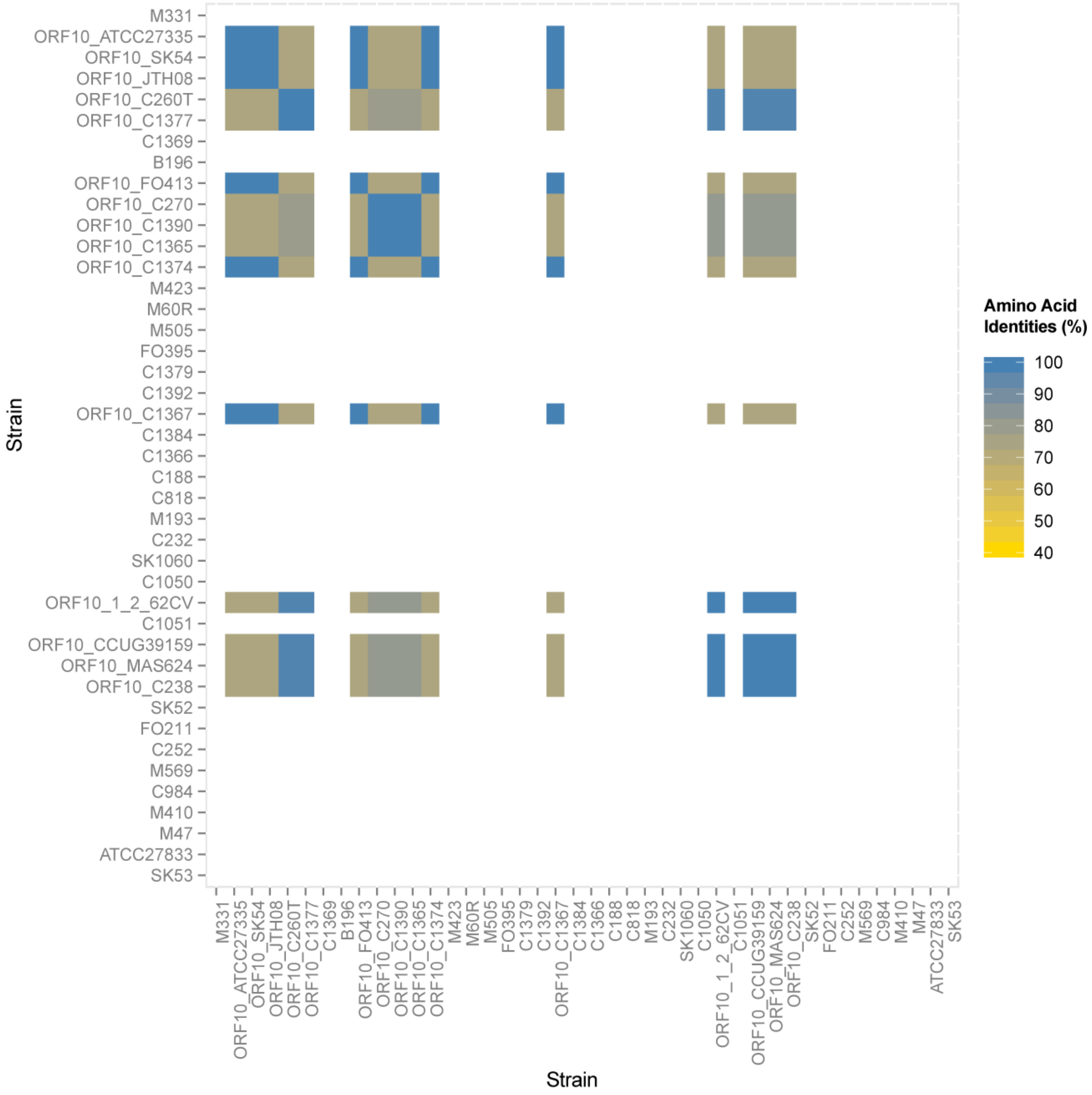

Figure S23: Amino acid identity of ORF 10 in *Streptococcus* Anginosus Group (SAG) strains. The strains are described in Table 1. Strains are ordered as shown in Figure 3.

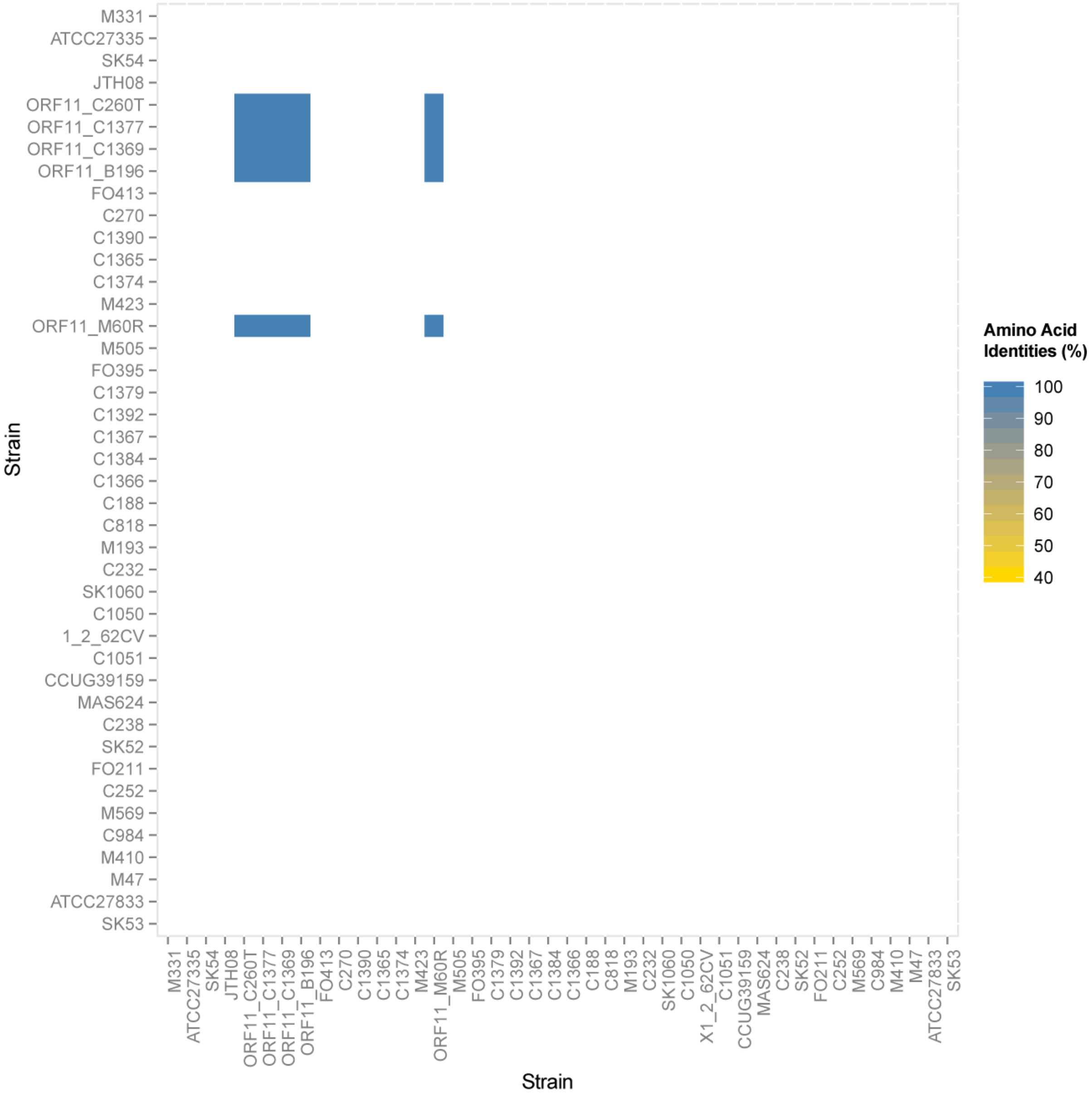

Figure S24: Amino acid identity of ORF 11 in *Streptococcus* Anginosus Group (SAG) strains. The strains are described in Table 1. Strains are ordered as shown in Figure 3.

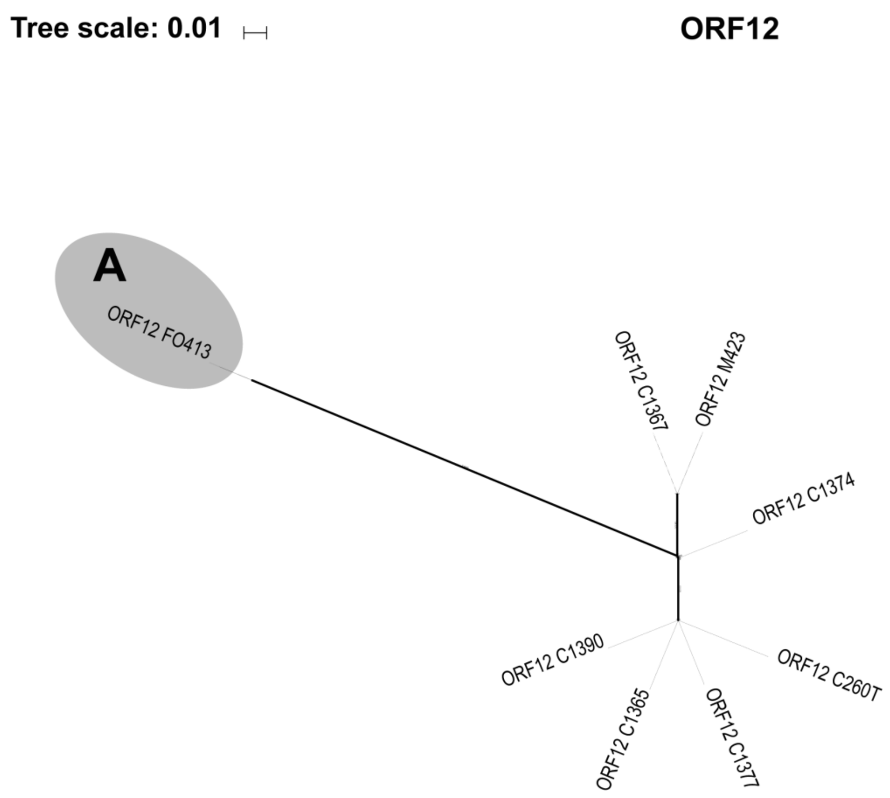

Figure S25: Phylogenetic tree of ORF 12 in the *Streptococcus* Anginosus Group (SAG). The tree was generated using FastTree (Price et al. 2009). SAG strains are labelled with descriptions available in Table 1.

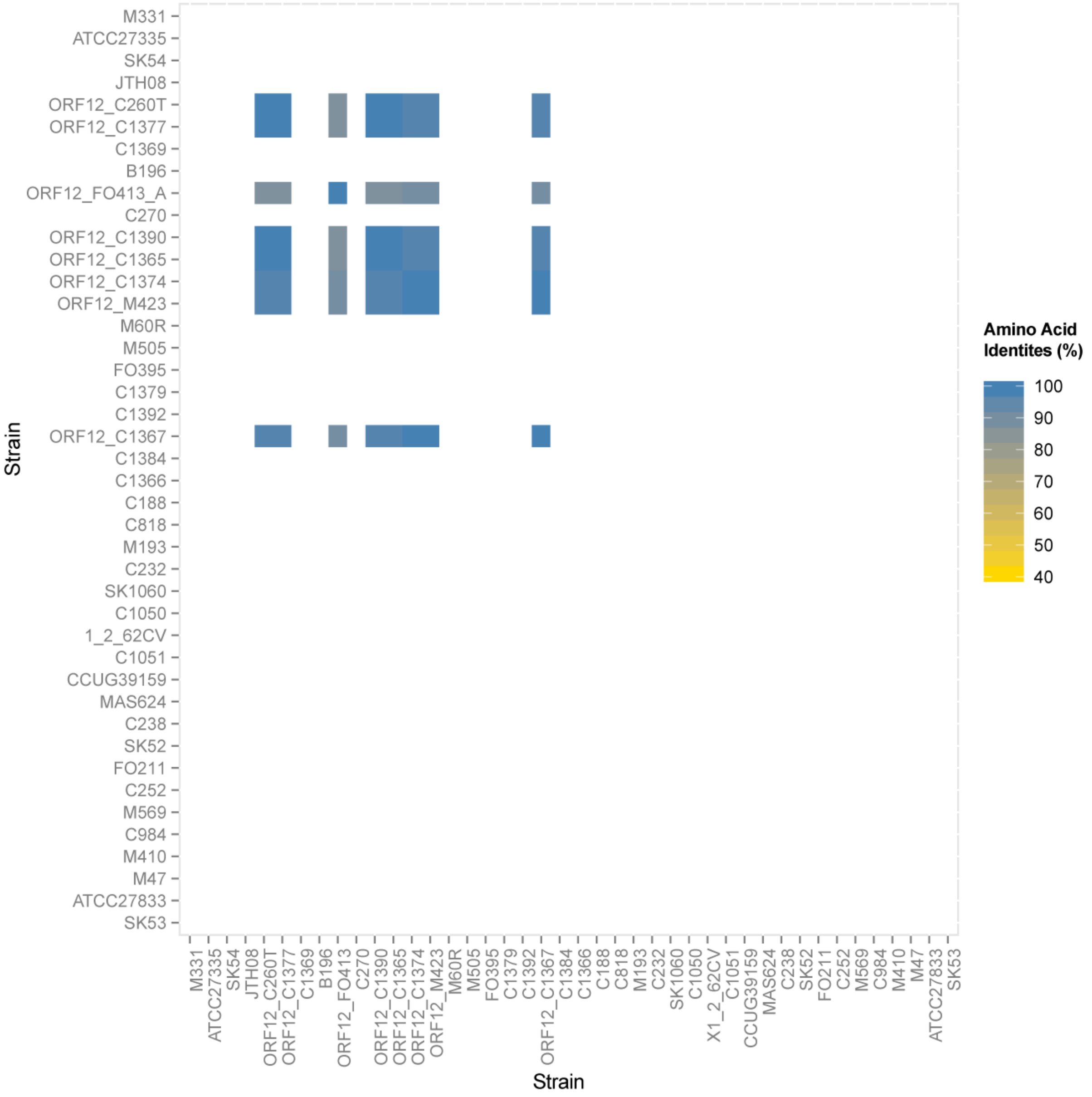

Figure S26: Amino acid identity of ORF 12 in *Streptococcus* Anginosus Group (SAG) strains. The strains are described in Table 1. Strains are ordered as shown in Figure 3.

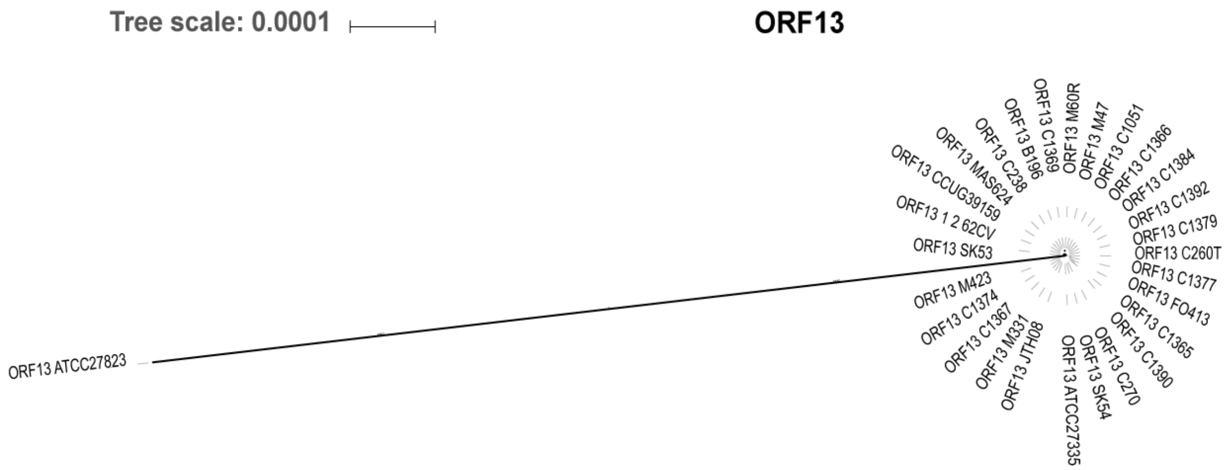

Figure S27: Phylogenetic tree of ORF 13 in the *Streptococcus* Anginosus Group (SAG). The tree was generated using FastTree (Price et al. 2009). SAG strains are labelled with descriptions available in Table 1.

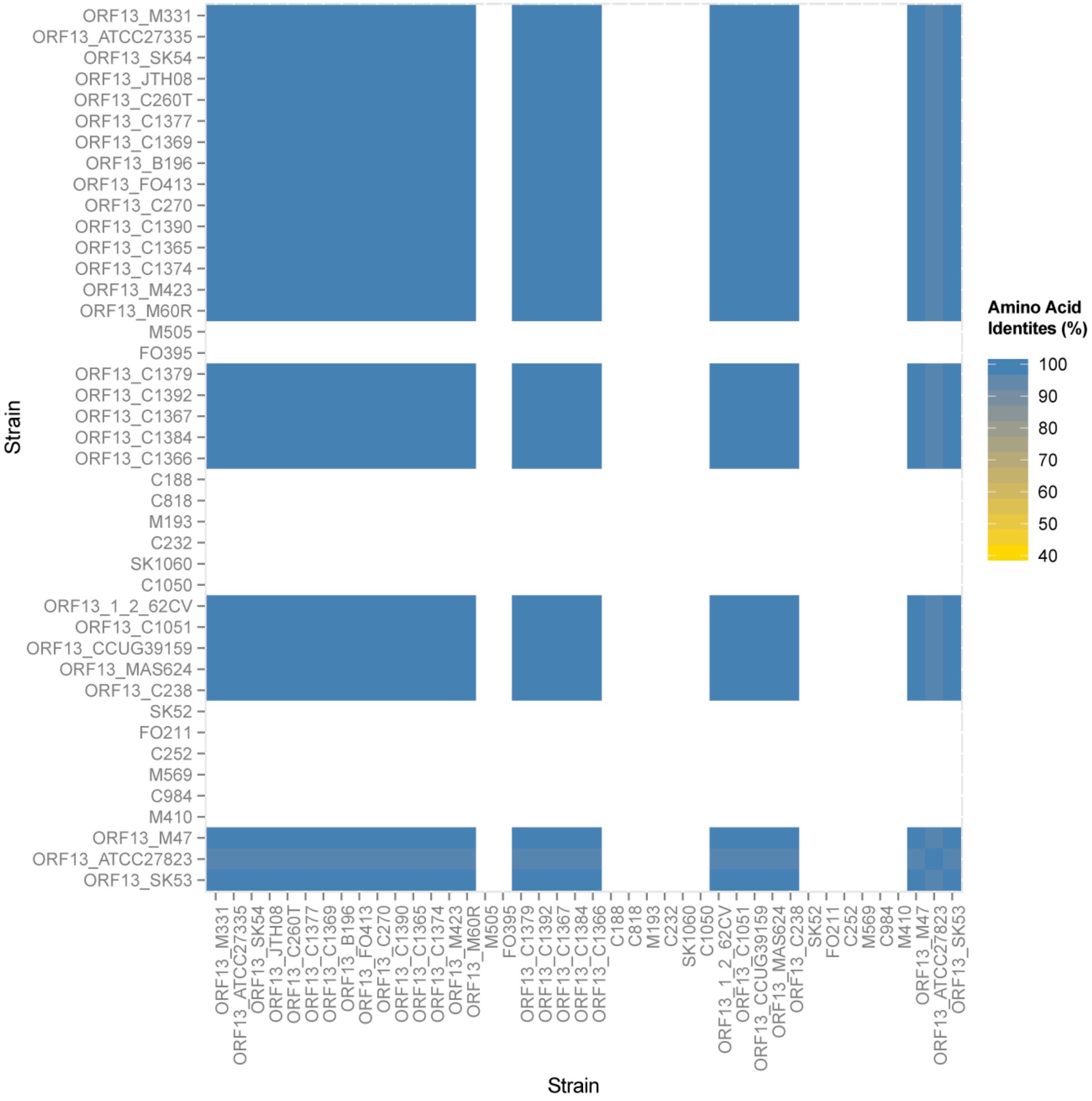

Figure S28: Amino acid identity of ORF 13 in *Streptococcus* Anginosus Group (SAG) strains. The strains are described in Table 1. Strains are ordered as shown in Figure 3.

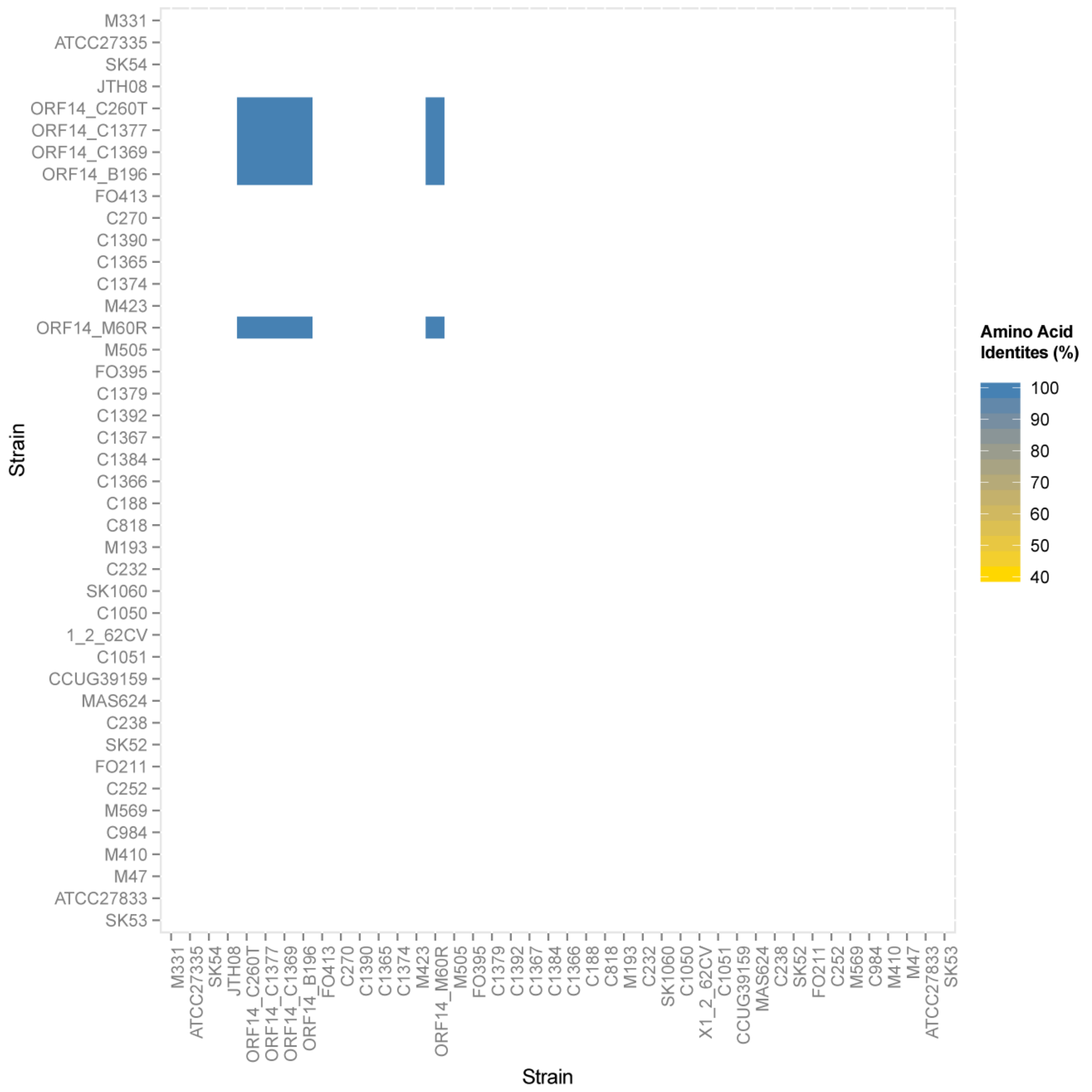

Figure S29: Amino acid identity of ORF 14 in *Streptococcus* Anginosus Group (SAG) strains. The strains are described in Table 1. Strains are ordered as shown in Figure 3.

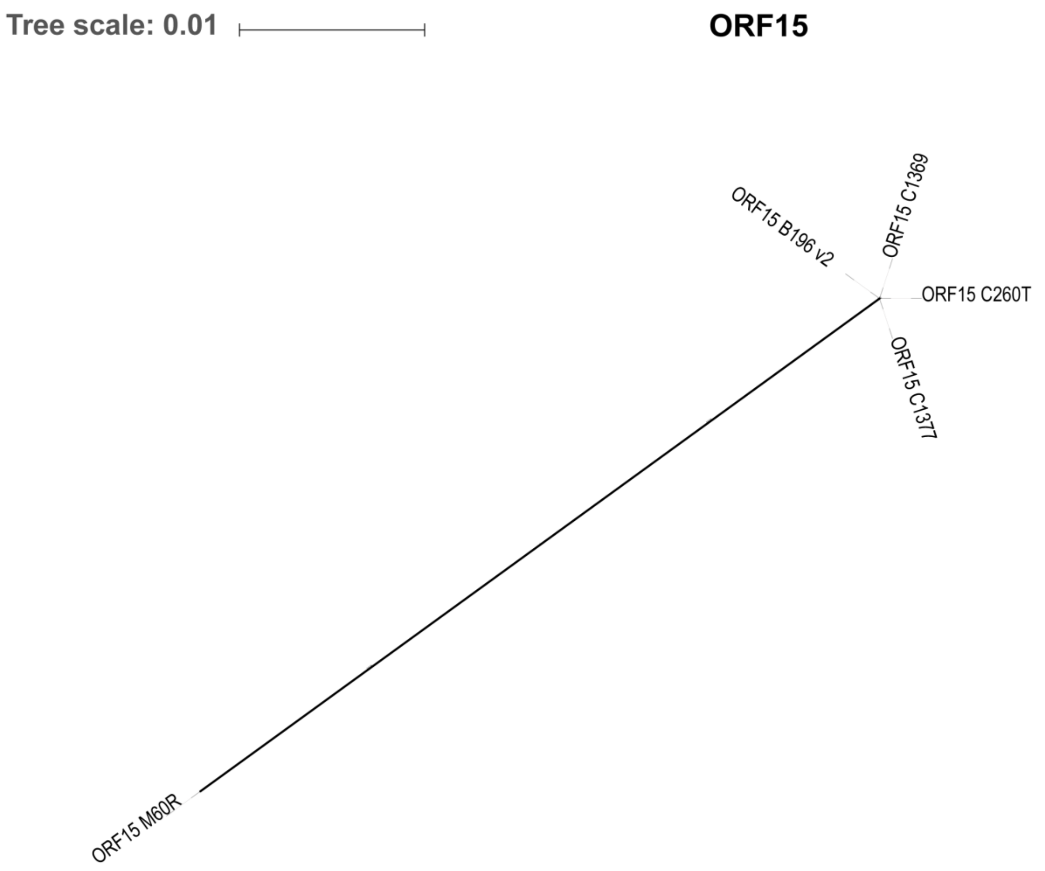

Figure S30: Phylogenetic tree of ORF 15 in the *Streptococcus* Anginosus Group (SAG). The tree was generated using FastTree (Price et al. 2009). SAG strains are labelled with descriptions available in Table 1.

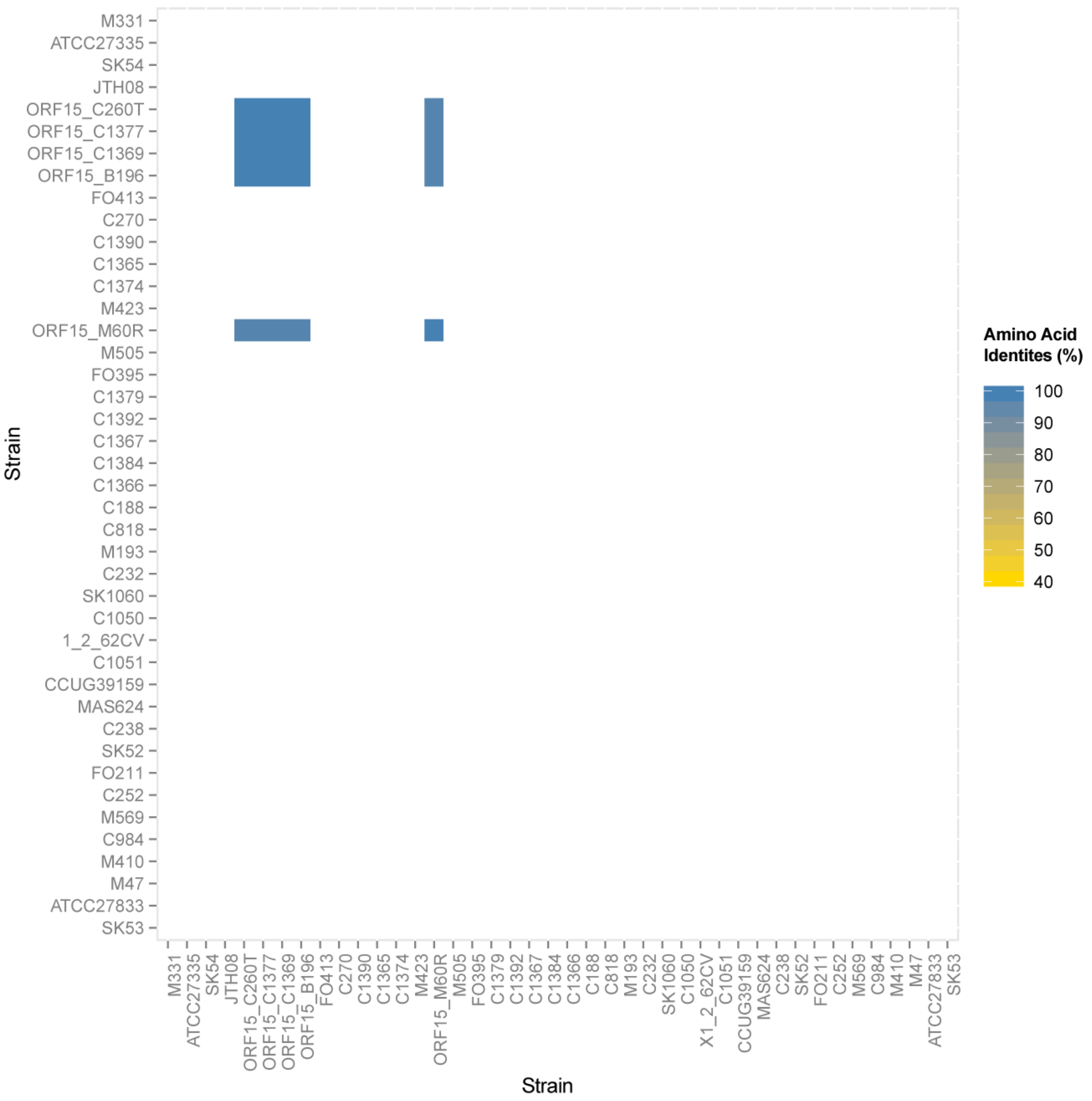

Figure S31: Amino acid identity of ORF 15 in *Streptococcus* Anginosus Group (SAG) strains. The strains are described in Table 1. Strains are ordered as shown in Figure 3.

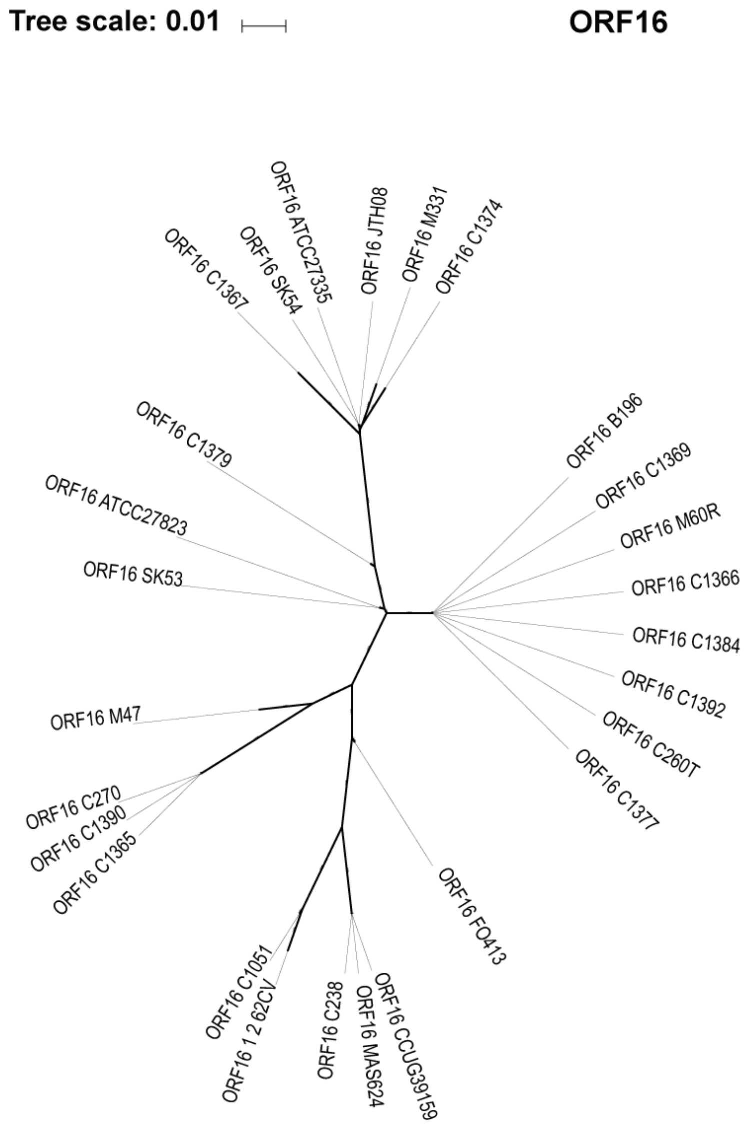

Figure S32: Phylogenetic tree of ORF 16 in the *Streptococcus* Anginosus Group (SAG). The tree was generated using FastTree (Price et al. 2009). SAG strains are labelled with descriptions available in Table 1.

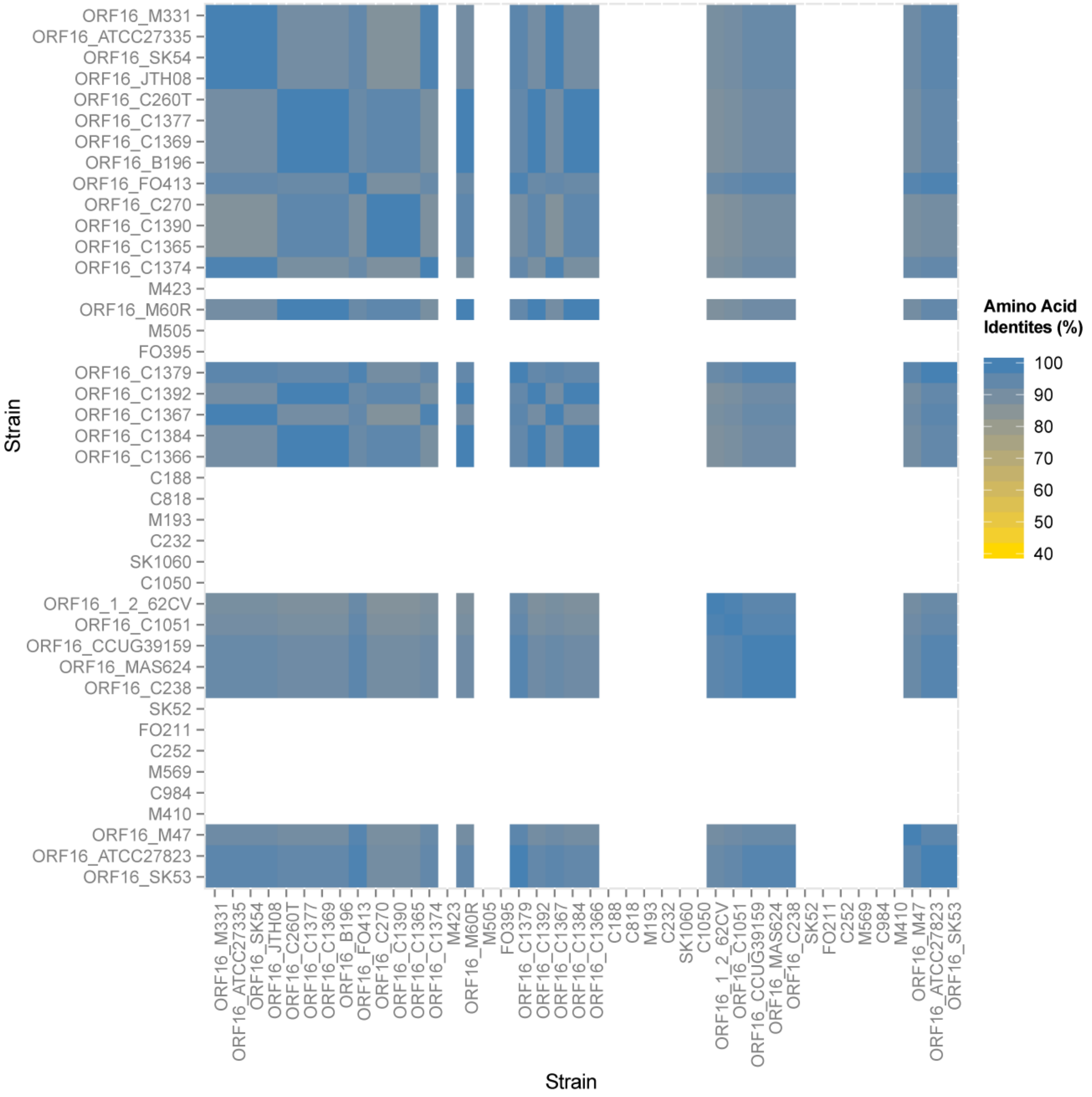

Figure S33: Amino acid identity of ORF 16 in *Streptococcus* Anginosus Group (SAG) strains. The strains are described in Table 1. Strains are ordered as shown in Figure 3.
